# Statistical evaluation of methods for identification of differentially abundant genes in comparative metagenomics

**DOI:** 10.1186/s12864-016-2386-y

**Published:** 2016-01-25

**Authors:** Viktor Jonsson, Tobias Österlund, Olle Nerman, Erik Kristiansson

**Affiliations:** Department of Mathematical Sciences, Chalmers University of Technology and University of Gothenburg, Gothenburg, SE-412 96 Sweden

**Keywords:** Environmental sequencing, Next generation sequencing, Categorical data analysis, Differential abundance, Receiver operating characteristic, False discovery rate

## Abstract

**Background:**

Metagenomics is the study of microbial communities by sequencing of genetic material directly from environmental or clinical samples. The genes present in the metagenomes are quantified by annotating and counting the generated DNA fragments. Identification of differentially abundant genes between metagenomes can provide important information about differences in community structure, diversity and biological function. Metagenomic data is however high-dimensional, contain high levels of biological and technical noise and have typically few biological replicates. The statistical analysis is therefore challenging and many approaches have been suggested to date.

**Results:**

In this article we perform a comprehensive evaluation of 14 methods for identification of differentially abundant genes between metagenomes. The methods are compared based on the power to detect differentially abundant genes and their ability to correctly estimate the type I error rate and the false discovery rate. We show that sample size, effect size, and gene abundance greatly affect the performance of all methods. Several of the methods also show non-optimal model assumptions and biased false discovery rate estimates, which can result in too large numbers of false positives. We also demonstrate that the performance of several of the methods differs substantially between metagenomic data sequenced by different technologies.

**Conclusions:**

Two methods, primarily designed for the analysis of RNA sequencing data (edgeR and DESeq2) together with a generalized linear model based on an overdispersed Poisson distribution were found to have best overall performance. The results presented in this study may serve as a guide for selecting suitable statistical methods for identification of differentially abundant genes in metagenomes.

**Electronic supplementary material:**

The online version of this article (doi:10.1186/s12864-016-2386-y) contains supplementary material, which is available to authorized users.

## Background

Metagenomics is the study of microorganisms by sequencing random pieces of their genomes directly from environmental and clinical samples [1, 2]. In contrast to many traditional methods in microbiology, metagenomics require no prior cultivation of individual isolates and entire communities can therefore be studied directly in their natural state [3]. The recent development of cost-efficient high-throughput DNA sequencing technologies has greatly increased the popularity and potential of metagenomics and it has become a key technique for the analysis of the human microbiome, its composition and connection to disease [4–6]. Environmental microbial communities are also extensively studied using metagenomics in order to assess their structure and diversity [7–9].

Metagenomics are often analyzed in a gene-centric approach where the individual genes are quantified in a process called binning [10]. After quality assessment of the raw sequence data, each fragment is matched against a reference database which typically consists of annotated genomes, contigs or a catalogue of genes. The relative abundance of each gene (bin) is then estimated by counting the number of matching fragments in relation to a reference value such as the total number of fragments in the sample. By comparing gene abundance between metagenomes, important differences in community structure, diversity and biological function can be identified. The identification of differentially abundant genes between metagenomes is however complex. Most metagenomes contain a high diversity of microorganisms which carries a vast number of different genes. The resulting count data is therefore high-dimensional with many thousands of genes quantified in a single sample. Metagenomic data is also plagued by high levels of biological and technical variability and the number of samples is often low (<10) [11–14]. Most metagenomes are also vastly undersampled and genes can therefore be represented by only a few, or even zero, DNA fragments. Thus, methods for statistical inference of metagenomic count data need to be robust to noise and have a high power to identify the truly differentially abundant genes. In addition, the high dimensionality can result in a large number of false positives and controlling the type I error as well as unbiased estimation of the false discovery rate is therefore vital.

A wide range of methods have been developed for identification of differentially abundant genes in metagenomic count data. XIPE-TOTEC, one of the first methods developed for this purpose, uses a permutation-based approach to estimate the median difference for each gene. Significance is calculated by comparing the estimated median to a null distribution generated by pooling the metagenomic samples [15]. Another early metagenome analysis tool was IMG/M which performs a test of the relative gene abundances using a Gaussian approximation under the null hypothesis [16, 17]. ShotgunFunctionalizeR is a software package for R containing several methods but has a focus on regression type approaches using generalized linear models [18]. MetaStats is based on a *t*-test where the *p*-values are derived from an empiric null distribution calculated by permuting the samples [19]. STAMP focuses on comparisons of pairs of metagenomes using Fisher’s exact test, but have recently been updated to also include other statistical procedures such as Welch’s *t*-test and the resampled t-statistic of MetaStats [20, 21]. LEfSe applies the non-parametric Kruskal-Wallis and Wilcoxon-Mann–Whitney tests to assess differences in gene abundance between groups and subgroups of metagenomes [22]. Another package for analysis of gene abundances is FANTOM which implements several parametric and non-parametric standard tests together with an easy-to-use graphical interface [23]. The more recently developed metagenomeSeq which uses a zero inflated Gaussian model to correct for bias caused by undersampling in combination of inference using the empirical Bayesian model implemented in Limma [24, 25]. Metagenomic data shows similarities to sequence-based transcriptomics and methods originally developed for analysis of RNA sequencing (RNA-seq) data have therefore been applied to identify differentially abundant genes, in particular edgeR [26, 27], DESeq2 [28, 29] and voom [30, 31]. Even though a wide range of methods have been suggested for the analysis of metagenomic data, there exists no comprehensive evaluation that investigates their performance and statistical properties under realistic settings.

In this paper we present a comparison of 14 methods for statistical analysis of metagenomic gene count data. Each method was assessed based on its statistical power to identify differentially abundant genes, its model assumptions and ability to control the false discovery rate. The methods were evaluated on data created by resampling and downsampling of real metagenomes which, in contrast to simulations from parametric distributions, results in more realistic settings where the structures of true gene count data are preserved. Our results revealed large differences in performance between the methods. The sample size, effect size and gene abundance greatly affected the ability to identify differentially abundant genes. Most methods showed skewed *p*-value distributions under the null hypothesis indicating non-adequate model assumptions. Most methods were able to control the false discovery rate but showed differences in the number of true positives detected. We conclude that no single method is optimal for all types of metagenomics datasets. The results presented in this study can therefore serve as a guide for selection of proper statistical methods for the analysis of metagenomic data.

## Results

### Sample size, effect size and gene abundance have a large impact on performance

**Fig. 1:**
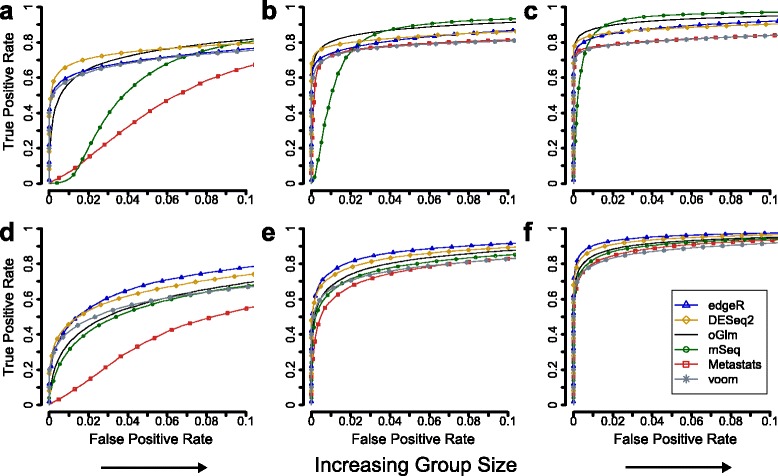
The performance of detecting differentially abundant genes increases for large group sizes. For each method, the receiver operating characteristics curve shows the true positive rate (y-axis) and the false positive rate (x-axis) at each position in the gene ranking list. Panels **a**-**c** show results for the Qin dataset and panels **d**-**f** show results for the Yatsunenko dataset. Group sizes of 3 + 3, 6 + 6 and 10 + 10 were included in the comparison and the effect size was fixed at a fold-change of 5. Each curve is based 100 resampled metagenomes. The methods included are edgeR, DESeq2, the overdispersed generalized linear model (oGLM), metagenomeSeq (mSeq), metastats and voom (see Additional file 2: Figure S1 for the additional eight methods)

Fourteen methods for identification of differentially abundant genes were evaluated on groups of metagenomes created by resampling from two datasets, one based on Illumina sequencing (Qin) and one from massively parallel pyrosequencing (Yatsunenko). Effects were introduced to 10 % of the genes using downsampling of fragments and the gene ranking performance was compared using receiver operating characteristics (ROC) curves and their corresponding area under curve up to a false positive rate of 0.05 (denoted AUC_0.05_, see Methods). Our results showed that the group size had a positive impact on the accuracy of gene ranking and the performance increased substantially when more samples were included (fold-change fixed to 5) (Table 1, Fig. 1, Additional file 1: Table S1). For the Qin dataset, no single method had the best performance for all investigated group sizes (Fig. 1a-c). At a group size of 3 + 3, DESeq2 had the highest performance with an AUC_0.05_ of 0.70 followed by edgeR (AUC_0.05_ of 0.64) and the overdispersed generalized linear model (OGLM) (AUC_0.05_ of 0.64) (Table 1). At larger group sizes, OGLM had the best performance with an AUC_0.05_ of 0.83 and 0.90 for 6 + 6 and 10 + 10 respectively. The corresponding numbers for DESeq2 were 0.80 and 0.86 and for edgeR 0.77 and 0.85. MetagenomeSeq had a low AUC_0.05_ at small group sizes (0.29 and 0.68 at 3 + 3 and 6 + 6 respectively), but at the largest group size the performance was second best after OGLM (AUC_0.05_ of 0.87). Voom and Metastats, which are both based on normal approximations, showed similar performances except at the smallest group size where Metastats performed poorly.

**Table 1:** The gene ranking performance at different group sizes for all 14 methods

AUC_0.05_	Data set 1: (Qin 2010)			Data set 2: (Yatsunenko 2012)		
Group Size	3 + 3	6 + 6	10 + 10	3 + 3	6 + 6	10 + 10
edgeR	0.64	0.77	0.85	0.55	0.80	0.92
DESeq2	0.70	0.80	0.86	0.53	0.78	0.90
OGLM	0.64	0.83	0.90	0.44	0.73	0.88
MetagenomeSeq	0.29	0.68	0.87	0.41	0.70	0.86
Metastats	0.19	0.74	0.79	0.18	0.66	0.84
Voom	0.63	0.74	0.78	0.48	0.70	0.83
Sqrt *t*-test	0.62	0.78	0.85	0.43	0.70	0.86
Log *t*-test	0.63	0.76	0.79	0.44	0.69	0.84
*t*-test	0.56	0.77	0.85	0.38	0.67	0.84
Welch *t*-test	0.47	0.74	0.83	0.32	0.62	0.82
WMW	-----	0.75	0.83	-----	0.66	0.84
Binomial	0.40	0.46	0.48	0.33	0.51	0.67
GLM	0.32	0.35	0.36	0.34	0.52	0.68
Fisher’s exact test	0.32	0.35	0.36	0.33	0.51	0.67

Each listed value is the normalized area under curve up until a false positive rate of 0.05. Higher values represent higher gene ranking performance. The results are calculated based on 100 resampled metagenomes. The Wilcoxon-Mann–Whitney test was not evaluated at the smallest sample size (3 + 3) due to lack of samples. The full area under curve (AUC) measurements are available in Additional file 1: Table S1

The performance of the methods was more consistent across group sizes when evaluated on metagenomes resampled from the Yatsunenko dataset (Fig. 1d-f) (Table 1). Here, edgeR had the highest performance in all group sizes with an AUC_0.05_ of 0.55, 0.80 and 0.92 for 3 + 3, 6 + 6 and 10 + 10 respectively. DESeq2 was a close second followed by OGLM and metagenomeSeq (mSeq). Voom was the third best at 3 + 3 but had the lowest performance of all method at 10 + 10. Metastats again performed poorly at low sample sizes but was close to the top performing methods at 10 + 10.

The ordinary Student’s *t*-test using a square-root variance stabilizing transform had a surprisingly high performance with an AUC_0.05_ of 0.86 for the Yatsunenko dataset and 0.85 for the Qin dataset at a group size of 10 + 10 (Table 1, Additional file 2: Figure S1). Interestingly, when the square-root transform was replaced with a log-transform, the performance decreased for most group sizes on both datasets. The non-parametric Wilcoxon-Mann–Whitney test showed a lower performance than the *t*-test. Finally, the Poisson generalized linear model, the Fisher’s exact test and the binomial test all had a consistently poor performance for all group sizes and datasets.

Next, the impact of the effect size was investigated for a fold-change of 3, 5 and 7. As expected, all methods performed better at larger effect sizes (Additional file 3: Figure S2, Additional file 4: Figure S3, Additional file 5: Table S2, Additional file 6: Table S3). For the Qin datasets, the best method (OGLM) had an AUC_0.05_ of 0.74, 0.83 and 0.86 for effect sizes 3, 5 and 7 respectively (group size fixed to 6 + 6). For the Yatsunenko datasets, edgeR had the highest performance with an AUC_0.05_ of 0.63, 0.80 and 0.87 for the three effect sizes. Altering the effect size did not substantially change the relative performance of the methods.

**Fig. 2:**
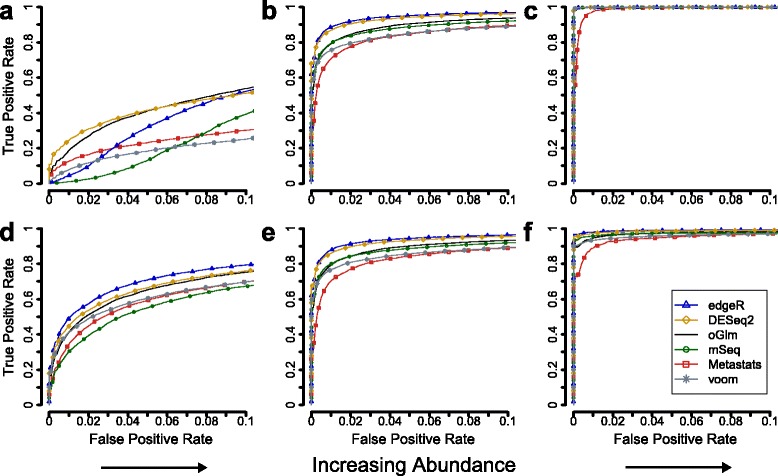
Gene abundance had a large impact on the performance to identify differentially abundant genes. For each method, the receiver operating characteristics curve shows the true positive rate (y-axis) and the false positive rate (x-axis) at each position in the gene ranking list. Panels (**a**-**c**) show results for the Qin dataset and panels (**d**-**e**) show results for the Yatsunenko dataset. The genes were stratified into three parts based on the average number of DNA fragments, i) ≤500, ii) 500–5000 and iii) >5000 for the Qin dataset and i) ≤10, ii) 10–50 and iii) >50 for the Yatsunenko dataset. The effect size was set to a fold-change of 5 and the group size fixed at 6 + 6 samples. Each curve is based 100 resampled metagenomes. The methods included are edgeR, DESeq2, the overdispersed generalized linear model (oGLM), metagenomeSeq (mSeq), metastats and voom (see Additional file 7: Figure S4 for the additional eight methods)

The power to identify differentially abundant genes is dependent on the number of observed DNA fragments. To investigate this effect, the genes were stratified into three roughly equally sized groups based on their average number of fragments (see Methods). This showed that the gene abundance had a considerable impact on the gene ranking performance. For the Qin dataset, all methods showed a poor ranking performance for genes with a low abundance (<500), where DESeq2 had the highest and metagenomeSeq the lowest AUC_0.05_, 0.31 and 0.05 respectively (Fig. 2a, effect and group size were fixed to 5 and 6 + 6 respectively). The ranking performance increased substantially for genes with higher abundance (500–5000 fragments, Fig. 2b, Additional file 7: Figure S4) where OGLM had the highest AUC_0.05_ at 0.93. At the highest abundance group (>5000 fragments), all methods generated excellent ranking of the differentially abundant genes with the only exception of the generalized linear model, the Fisher’s exact test and the binomial test (Table 2, Fig. 2c, Additional file 7: Figure S4, Additional file 8: Table S4). Analogously to the Qin dataset, all methods had a poor performance for the low abundant genes in the Yatsunenko dataset (<50 fragments) which increased substantially at higher abundance (50–500 and >500) (Fig. 2d-f). EdgeR was the best method in all three categories with an AUC_0.05_ of 0.58, 0.90 and 0.99 (Table 2). MetagenomeSeq (mSeq) showed again a poor performance for low abundant genes (AUC_0.05_ of 0.40 at <50 fragments) but was the third best method when the abundance increased (AUC_0.05_ of 0.97 at >500 fragments). For the low abundant genes, all methods showed a lower performance for the Qin dataset compared to the Yatsunenko dataset, even though the cutoff was tenfold higher (<500 and <50 for Qin and Yatsunenko respectively).

**Table 2:** The gene ranking performance at different gene abundances for all 14 methods

AUC_0.05_	Data set 1: (Qin 2010)			Data set 2: (Yatsunenko 2012)		
Mean Abundance:	<500	500–5000	>5000	<10	10–50	>50
edgeR	0.15	0.89	1.00	0.58	0.90	0.99
DESeq2	0.31	0.91	1.00	0.54	0.89	0.98
OGLM	0.28	0.93	1.00	0.51	0.83	0.96
MetagenomeSeq	0.05	0.92	1.00	0.40	0.83	0.97
Metastats	0.17	0.82	0.97	0.45	0.75	0.91
Voom	0.12	0.84	1.00	0.49	0.80	0.94
Sqrt *t*-test	0.21	0.88	1.00	0.48	0.80	0.95
Log *t*-test	0.17	0.86	1.00	0.45	0.80	0.95
*t*-test	0.23	0.86	0.99	0.43	0.77	0.94
Welch *t*-test	0.20	0.81	0.98	0.39	0.72	0.91
WMW	0.21	0.84	0.97	0.43	0.75	0.92
binomial	0.15	0.56	0.60	0.31	0.68	0.82
GLM	0.15	0.48	0.31	0.32	0.68	0.82
Fisher’s exact test	0.15	0.48	0.31	0.31	0.68	0.82

Each listed value is the normalized area under curve up until a false positive rate of 0.05. Higher values represent higher gene ranking performance. The results are calculated based on 100 resampled metagenomes. The Wilcoxon-Mann–Whitney test was not evaluated at the smallest sample size (3 + 3) due to lack of samples. The full area under curve (AUC) measurements are available in Additional file 8: Table S4

Most methods accurately estimated the effect size in the Qin dataset (See Additional file 9: Figure S5). For the Yatsunenko dataset however, several methods, including DESeq2, metagenomeSeq, MetaStats and voom, showed underestimated effect sizes. This was in contrast to edgeR and OGLM which both produced unbiased estimates for both datasets. The standard deviation of the estimated effect size decreased, as expected, for all method as the group size increased.

### Most methods have a biased *p*-value distribution under the null hypothesis

**Fig. 3:**
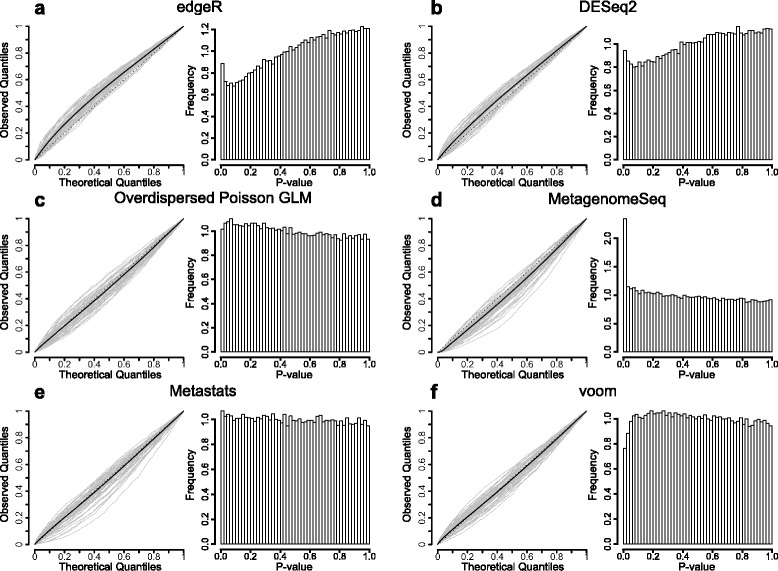
Most methods have a biased *p*-value distribution under the null hypothesis. The *p*-value distributions on the Qin dataset with no added effect and a group size of 6 + 6 averaged over 100 resampled data sets. For the quantile-quantile-plots, each grey line represent a resampled metagenome, the solid black line represents the average value and the dotted line the line with slope one corresponding to a uniform *p*-value distribution. The panels correspond to edgeR (**a**), DESeq2 (**b**), overdispersed Poisson GLM (**c**), metagenomeSeq (**d**), metastats (**e**) and voom (**f**). (See Additional file 10: Figure S6 for the additional eight methods)

**Fig. 4:**
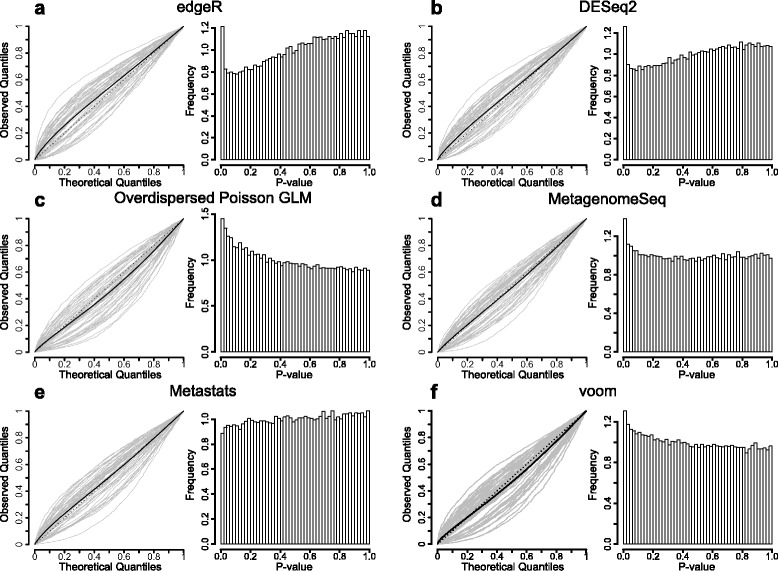
Quantile-quantile plots and histograms for the second data set under the null hypothesis. The *p*-value distributions on the Yatsunenko dataset with no added effect and a group size of 6 + 6 averaged over 100 resampled metagenomes. For the quantile-quantile-plots, each grey line represent a resampled metagenome, the solid black line represents the average value and the dotted line the line with slope one corresponding to a uniform *p*-value distribution. The panels correspond to edgeR (**a**), DESeq2 (**b**), overdispersed Poisson GLM (**c**), metagenomeSeq (**d**), metastats (**e**) and voom (**f**). (See Additional file 11: Figure S7 for the additional eight methods)

Unbiased estimation of *p*-values under the null hypothesis is essential to control the type I error rate. We therefore used resampled metagenomes without added effects to investigate the *p*-value distributions for all 14 methods (see Methods). The majority of the methods showed biased *p*-values with distributions skewed towards either low or high values (Figs. 3 and 4, Additional file 10: Figure S6 and Additional file 11: Figure S7). EdgeR and DESeq2 both had conservative *p*-values (Figs. 3a, b, 4a, b) while the *p*-values for OGLM were too optimistic (Figs. 3c, 4c). These trends were consistent between the two datasets. MetagenomeSeq demonstrated too optimistic *p*-value distribution and this bias was more pronounced for the Qin datasets where a large proportion of the genes had very small *p*-values (Figs. 3d, 4d). Metastats had the most uniform *p*-value distribution (Fig. 3e, 4e) while voom exhibited slightly conservative *p*-values for the Qin dataset (Fig. 3f) but had too optimistic *p*-values for the Yatsunenko dataset (Fig. 4f). All tests based on t-statistics showed a unimodal *p*-value distribution where the variant using the square-root transformation was most uniform (Additional file 10: Figure S6, Additional file 11: Figure S7). Finally, the Poisson generalized linear model, the Fisher’s exact test and the binomial test all had extremely optimistic *p*-values indicating that these methods will likely produce a high number of false positives.

### Several methods are able to control the FDR but their power differ

**Fig. 5:**
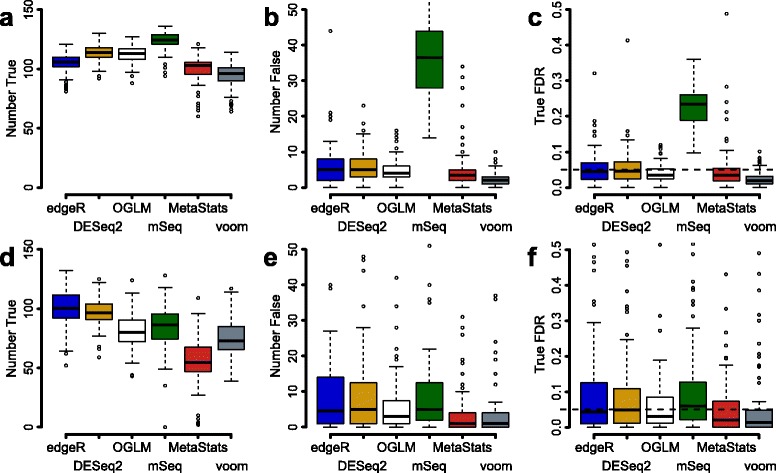
Most methods can control the false discovery rate at predefined level. The figure shows boxplots of the number of true positives (panel **a**, **d**), the number of false positives (panel **b**, **e**) and achieved true FDR (panel **c**, **f**) at a cutoff of 0.05 estimated FDR. Panels (**a**-**c**) show results for the Qin dataset and panels (**d**-**f**) show results for the Yatsunenko dataset. The group sizes were set to 6 + 6 and the effect size to 5. The results were based on 100 resampled metagenomes. The included methods are edgeR, DESeq2, the overdispersed generalized linear model (oGLM), metagenomeSeq (mSeq), metastats and voom (see Additional file 12: Figure S8 for the additional eight methods)

The ability to control the false discovery rate (FDR) was analyzed for each method by counting the number of true and false positives at an estimated FDR of 0.05. (Fig. 5, Additional file 12: Figure S8). For the Qin dataset, metagenomeSeq detected the highest number of true positives (123) in median, followed by DESeq2 (112), OGLM (112) and edgeR (104) (effect and group size fixed to 5 and 6 + 6 respectively) (Fig. 5a). However, metagenomeSeq produced a high number of false positives (37) while the numbers were substantially lower for DESeq2 (5), OGLM (4) and edgeR (5). Consequently, metagenomeSeq failed to control the false discovery rate and at an estimated FDR of 0.05 the true median FDR was 0.22. DESeq2, OGLM and edgeR were able to control the false discovery rate and at the 0.05 cut-off, the estimated FDR were 0.044, 0.035 and 0.040 respectively. For the Yatsunenko dataset, edgeR and DESeq2 identified the highest number of true positives (100) followed metagenomeSeq (86) and OGLM (80) (Fig. 5d). All these methods maintained a low number of false positives (4–5) (Fig. 5e) resulting in a true FDR at or below 0.05 for all methods (Fig. 5f). The t-statistics showed a slightly conservative FDR estimated for both the Qin and Yatsunenko datasets (Additional file 13: Table S5). The Poisson generalized linear model, the Fisher’s exact test and binomial test completely failed to control the false discovery rate with a too high proportion of false positives.

## Discussion

In this study, we evaluated the performance of 14 methods for the identification of differentially abundant genes between two groups of metagenomes. The statistical power, the uniformity of the *p*-values under the null hypothesis and the ability to control the false discovery rate were investigated using resampling of two large human gut metagenomic datasets, one based on Illumina sequencing (Qin) and on massively parallel pyrosequencing (Yatsunenko). Our results showed that the group size, effect size and gene abundance all had a large impact on the gene ranking performance of all methods. DESeq2, edgeR and the overdispersed Poisson GLM (OGLM) had the best overall performance, but their results differed between the investigated data sets and conditions. DESeq2 and OGLM had the highest performance on the Illumina dataset while edgeR was the best method on the dataset sequenced by massively parallel pyrosequencing. In addition, edgeR and OGLM had the most accurate estimation of the effect size, while DESeq2 produced biased estimates for the Yatsunenko dataset. DESeq2 and edgeR were originally developed for identification of differentially expressed genes in RNA-seq data. Both methods apply a negative binomial distribution where the gene-specific overdispersion is robustly calculated by a shrinkage estimator modelled by an empirical Bayes approach [26, 28]. For RNA-seq data, this has been shown to be highly advantageous when few samples are available and our evaluation shows that this is also true for metagenomic counts [32, 33]. However, at larger group sizes in the Qin dataset, OGLM had a higher performance than both DESeq2 and edgeR. In contrast, OGLM is a quasi-likelihood based method that assumes a Poisson distribution where the gene-specific overdispersion is introduced by scaling the gene abundance [18, 34]. Even though OGLM does not use any shrinkage approach to estimate the gene-specific overdispersion, it still had the highest performance for group sizes 6 + 6 and 10 + 10. This suggest that the underlying empirical Bayes models of DESeq2 and edgeR may not be fully optimal for all forms of count data in metagenomics and thus not always the preferable choice for identification of differentially abundant genes.

Another method that overall performed satisfactorily was metagenomeSeq, which is specifically developed for handling the high number of zero observations encountered in metagenomic data. MetagenomeSeq uses a log-transformation (*log*_*2*_*(y*_*ij*_ *+ 1)*) followed by correction for zero-inflation based on a Gaussian mixture model [24]. Inference is done after transformation using a normal-inverse gamma empirical Bayes model which moderates the gene-specific variance estimates [25]. Interestingly, our results show that the *t*-test using an identical log-transform had a higher performance in many of the testing conditions, especially for low abundant genes and small group sizes. This suggests that the correction for zero-inflation applied in metagenomeSeq may be disadvantageous under certain conditions and thus not recommended. However, it should be underlined that metagenomeSeq was primarily designed for inference of taxonomic composition using counts from amplicon sequencing (*e.g.* 16 s RNA) where the average number of counts and the number of samples typically is higher [24]. This is also confirmed by our results which show that metagenomeSeq has a substantially higher performance for genes with high abundance and at larger group sizes.

Gene count distributions have a non-trivial dependence between their mean and variance. This can negatively affect methods that do not specifically describe this dependence, such as methods based on Gaussian approximations. Variance stabilizing transformations can be used to decouple this dependence [35] and thereby increase the gene ranking performance significantly. The choice of variance stabilizing transformation is however dependent on the underlying distributional assumptions which are, for high-dimensional data, often hard to assess. We therefore evaluated the performance for two common transformations (square-root and log) and our results showed that the area under curve was higher for the square-root transformation than for the log-transformation for both investigated datasets. In fact, for large group sizes in the Qin dataset, applying a log-transform actually resulted in worse performance in comparison to non-transformed data. Thus, selecting an appropriate transformation has large impact on the statistical power of finding differentially abundant genes. Furthermore, the *t*-test with the square-root transform had a higher performance than the non-parametric Wilcoxon-Mann–Whitney test, even at a group size of 10 + 10. In contrast to the *t*-test, the Wilcoxon-Mann–Whitney test is less dependent on the underlying distributional assumptions but is vulnerable to ties [36]. Since observations with zero counts are common in metagenomic data it may explain the surprisingly low power [24]. Moreover, the Poisson generalized linear model, the Fisher’s exact test and the binomial test exhibited the worst performance under all tested conditions. These methods use, explicitly or implicitly, the group-wise pooled counts for inference without any estimate of the between sample variability. The low performance of these methods is thus due to their inability to correctly discriminate between overdispersion and effect. This further underlines the importance of proper modelling and estimation of the gene-specific variability for achieving a high statistical power in metagenomic analysis.

The *p*-values for non-differentially abundant genes should ideally be uniformly distributed between zero and one [37]. Deviation from the uniform distribution is an indication of wrong model assumptions and can result in too many false positives and incorrectly estimated false discovery rates. When applied to resampled metagenomes where all genes satisfied the null hypothesis of not being differentially abundant, most methods showed skewed *p*-value distributions with either too optimistic or too conservative values. MetaStats, which estimates the significance using permutations, was the only method able to produce in average unbiased *p*-values. This demonstrates the advantage of using empirically derived null distributions for controlling the type I error rate. In contrast, metagenomeSeq, generated highly biased *p*-values under the null hypothesis which resulted in a large number of false positives and a too optimistic false discovery rate. This is also in line with a recent evaluation of metagenomeSeq on count data of operational taxonomic units which demonstrated a high false positive rate [38]. Furthermore, the variability between individually resampled metagenomes was substantial and all methods produced both over- and under-estimated *p*-values for both datasets. This suggest a data heterogeneity where individual samples have substantial distributional differences that none of the methods can describe satisfactorily. Modelling of the sample-specific variability and between-sample correlations in large-scale transcriptomics have previously been shown to substantially reduce the bias of *p*-values under the null hypotheses and analogous models should be pursued in metagenomics in order to ensure a reliable estimation of the type I error rate [39, 40].

Calculation of the FDR is a common way to control the error rate in multiple testing of high-dimensional data [41]. Correct and unbiased estimation of the FDR is dependent on the model assumptions under both the null and alternative hypotheses and is vital for reliable downstream biological interpretation [42]. The majority of methods in this study were able to control the true FDR at the specified estimated FDR cut-off. This was however not true for metagenomeSeq which exhibited a too high proportion of false positives. The Poisson generalized linear model, the Fisher’s exact test and the binomial test were completely unable to control the FDR and returned a large number of false positives. Applying these methods to metagenomic data is thus not recommended and may lead to erroneous biological conclusions.

Two independent datasets were used as a basis for the evaluation; one generated using the Illumina platform (Qin) and one using massively parallel pyrosequencing (Yatsunenko). All methods had a slightly higher gene ranking performance for the Qin dataset, partially due to the substantially higher sequencing depth. However, all methods also showed a reduced performance for the low abundant genes in Qin dataset compared to the Yatsunenko dataset. This difference was substantial (Table 2), especially considering that the cut-off for the lowest abundant genes was ten-fold higher (<500 for Qin and <50 for Yatsunenko). The discrepancy for the low abundant genes in the two datasets can be partially explained by the higher variability caused by the large proportion of genes with zero counts available in the Qin dataset (Additional file 14: Figure S9). This is likely a consequence of the binning process typically applied for short read data where the reference database is first assembled *de novo*. Genes represented by a low number of fragments are often hard to assemble and may therefore be completely missing from the reference database, resulting in observations with zero fragments. Zero-inflation is known to result in overestimation gene-specific variability and will thus cause in an overall reduction in the power for identification of differentially abundant genes. Refined binning strategies using comprehensive reference databases, such as gene catalogue or fully sequenced genomes from a large collection of isolates, may result in a more accurate representation of the metagenome and thereby reduce the number of zero count observations [43, 44]. However, these strategies are only be applicable to well-studied microbial communities such as the human microbiome. Thus, development of new statistical methods that show a higher robustness against zero-inflation will be vital in order to maintain a high statistical performance in all forms of metagenomic studies.

Simulated metagenomes are dependent on the underlying assumptions and the specific parametric distribution used to draw gene counts. Comparisons using simulated data are therefore, in essence, subjective and may greatly favor methods with assumptions close to those used for the data generation. In this study, the performance of the statistical methods was evaluated on artificial datasets created by resampling of two real metagenomic datasets. In contrast to simulation from parametric distributions, resampling preserve many of the features of real metagenomic data, such as the underlying read distributions with its technical and biological variability and the gene-gene correlation which can have a large impact on estimation of the false discovery rate [45]. Effects were introduced in the data by a downsampling strategy where individual DNA fragments were randomly and independently removed from the dataset. It should be noted that our resampling approach works similar to an experimental randomization procedure in the sense that the effects added by downsampling are not systematically co-varying with other non-modeled factors for example, host age, life style or genetics. In a real experimental setup factors may covariate making inference even harder. However, we still argue that in contrast to simulating from a parametric distribution, our setup with resampled artificial metagenomes generates more realistic count data which leads to a more objective comparison of the statistical methods. Furthermore, the methods included in this study were run using their recommended normalization techniques (see Methods). However, normalization of metagenomic data has previously been shown to have a substantial impact on the analysis [38, 46] and a wide range of different techniques has so far been developed [24, 47, 48]. Since the methods included in this study are based on different distributional assumption is it also likely that they perform optimally in combination with different types of normalization. Further studies are therefore needed to identify which normalization strategies that should be combined with the different statistical methods in order to achieve maximum performance for identification of differentially abundant genes in metagenomic data.

## Conclusions

Statistical inference in metagenomics is challenging due to high levels of biological and technical variability in combination with high dimensionality of the count data and the few samples that are typically present. In this study 14 methods for identification of differentially abundant genes were evaluated. Our results showed that group size, effect size and gene abundance greatly affected the performance and no single method was best under all investigated conditions. DESeq2, the overdispersed Poisson generalized linear model and edgeR had all an overall satisfactory performance and are therefore suitable methods for inference of metagenomic gene count data. Our results also showed that methods that do not correctly capture the between-sample variability have a very low performance and should be avoided. The results presented in this paper may thus serve as a guide for the design of future metagenomic experiments and as suggestions for appropriate statistical methods to use in the analysis of gene count data.

## Methods

### Included methods

We assume that the metagenomic data is organized in a table of counts with *n* rows and *m* columns. The *n* rows correspond to bins, which typically represents gene families, functional groups or single genes. For consistency, we will in this paper use the word gene to represent all these possibilities. The *m = m*_*1*_ *+ m*_*2*_ columns represent metagenomes from two conditions containing *m*_*1*_ and *m*_*2*_ samples respectively.

Fourteen methods for identification of differentially abundant genes in metagenomics were selected to be included in the evaluation. MetagenomeSeq version 1.6 was applied to the data using default parameters [24]. MetaStats from the metagenomeSeq R-package 1.6 were run with the number of permutations increased from the default 1000 to 10000 [19]. EdgeR, from version 3.6.8 of the edgeR package, and voom, implemented in the limma R-package version 3.20.9, were run with default parameters [30, 49, 50]. DESeq2 version 1.4.5 was applied with the filters for outliers and genes with low abundance disabled [28].

The Poisson generalized linear model (GLM) and the overdispersed Poisson generalized linear model (OGLM) was implemented according to the R-package shotgunFunctionalizeR version 1.2-9 [18]. The Fisher’s exact test was implemented as a gene-specific 2 × 2 contingency table pooling fragments from the samples within each group. The table was constructed from the number of fragments matching and not matching the gene (rows) for the two groups (columns) [21] and the *p*-values were calculated using the fisher.test() function in R 3.1.1 [51]. The two-sided binomial test was implemented as described in [18]. Student’s and Welch’s *t*-test were implemented using the t.test() function in R. The t-tests were applied to the normalized counts with and without two common used variance stabilizing transforms, i) *y*^*1/2*^ and ii) *log(y + 1)* [35]. The Wilcoxon-Mann–Whitney test (WMW) was implemented using the wilcox.test() function in R. The *p*-values were derived based on permutations when no ties were present and on normal approximations when ties were present [52].

Each method was run with it’s recommended between sample normalization technique. MetagenomeSeq and metaStats used cumulative sum scaling [24], EdgeR and voom used TMM (Trimmed mean of M-values) [53] and DESeq2 used the median of rations [54]. The remaining methods all used the total number of binned fragments per sample as normalization factors, either by dividing the observed counts by the column total or providing the column totals as scaling factors.

### Resampling of gene count data

The statistical methods were evaluated on data created by resampling two large metagenomic datasets. The first dataset (Qin) consists of the fecal metagenomes from 124 individuals [55] and was sequenced using the Illumina platform resulting in a read length of 75 bases and, in average, 62.5 million reads per sample. The raw reads for 124 samples and individual-specific assembled contigs were retrieved from http://gutmeta.genomics.org.cn/. The contigs were annotated based on the TIGRFAM database, release 13.0 [56] using HMMER 3 [57] with a domain E-value cutoff of 10^−10^. The raw reads were binned and quantified against the assembled contigs using Tentacle v0.1 [58] using FASTX Toolkit v 0.0.13.2 for quality control (fastq_quality_filter, "-q 10 -p 50"; fastq_quality_trimmer "-t 1 -l 0") and pBLAT v.35 for read mapping [59] ("-threads 32 -minIdentity 90 -out = blast8"). Samples from the same individual were pooled after binning. Four individuals (MH0028, MH0037, MH0081, V1.CD.8) were excluded due to the low total counts. The second dataset (Yatsunenko) consists of 110 fecal metagenomes and was sequenced using massively parallel pyrosequencing resulting in an average read length of 341 bases and an average number of reads 155,890 [5]. The data was retrieved from the MG-RAST database [60], http://metagenomics.anl.gov/linkin.cgi?project=98. The reads were binned by translating them in the six possible frames and aligning the peptide sequences directly to gene models in the TIGRFAM database [56] using HMMER 3 [57] with an e-value cutoff of 10^−10^.

Resampled metagenomic data was created as follows. First two equally sized groups were randomly selected from one of the datasets (Qin or Yatsunenko) without replacement. All individuals, rather than a homogenous subset, were used in the resampling in order to capture the full variability present in the datasets. Next, 10 % of the genes were randomly selected to be differentially abundant and an effect was added by down-sampling of the reads. For these genes, the observed counts *y*_*ij*_ within one group (randomly selected) were replaced with new values y^*^_ij_ sampled a binomial distribution with parameters *y*_*ij*_ and *1/q* where *q* is the effect size parameter (fold-change). Downsampling can only be applied to a gene with non-zero counts and genes with i) zeros in more than 75 % of the samples or ii) an average abundance <3 in the sampled metagenomes were therefore excluded from the analysis. The average number of genes in the resampled datasets was after filtering 3029 and 2150 for the Qin and Yatsunenko datasets respectively. To assess the effect on the filtering, we performed an analysis on unfiltered data (effect size 5, group size 6 + 6) which is available in Table S6, Additional file 15.

Evaluation of the performance for low, intermediate and high abundant genes was done by stratifying the resampled data into three disjoint parts based on the average number of fragments. For the Qin dataset, these parts were defined as i) average abundance <500 fragments, ii) average abundance between 500 and 5000 and iii) average abundance >5000. For the Yatsunenko dataset the parts were defined as i) average abundance <50, ii) average abundance between 50 and 500 and iii) average abundance >500. The stratification was performed independently for each resampled dataset prior to adding effects. Each method was applied to the full data set but the results were compared within each abundance category.

### Ranking genes based on differential abundance

Receiver operating characteristic (ROC) curves [61] were used to visualize the performance of the methods. Each method was applied to each resampled dataset creating gene lists sorted according to increasing *p*-values. At each gene rank, the true positive rate (TPR) and false positive rate (FPR) were calculated for genes above that rank. The procedure was repeated 100 times and a consensus ROC curve was calculated using point-wise vertical averaging for fixed FPR values. Area under curve (AUC) was used to summarize the overall performance. In addition, the AUC up to a false positive rate cutoff of 0.05 (AUC_0.05_) was calculated focusing especially on the performance of the top of the ranking lists. The AUC_0.05_ was normalized by 0.05 to generate a value between 0 and 1. All AUC estimates were calculated using the trapezoid integral under the corresponding ROC curve.

### Distribution of *p*-values under null and FDR estimation

The distribution of method specific nominal *p*-values under the null hypotheses was examined using histograms and quantile-quantile plots (qq-plots). The qq-plots were created by first generating 100 resampled metagenomic datasets without adding differentially abundant genes. Each resampled dataset was down-sampled by randomly selecting exactly 1500 genes to make them comparable and an average qq-line was then calculated by taking the mean across each quantile. The *p*-value histograms were generated by pooling the *p*-values from the resampled datasets.

The estimated FDR was calculated by the Benjamini and Hochberg estimator,

$$ \widehat{FDR}(k)=\frac{np(k)}{k}, $$

where *p(k)* is the *p*-value at rank *k* and *n* is the total number of genes (using p.adjust in R 3.1.1) [41]. The true FDR was calculated from the rankings of each method, i.e.

$$ FDR(k)=\frac{\mathrm{Number}\ \mathrm{of}\ \mathrm{false}\ \mathrm{calls}\ \mathrm{up}\ \mathrm{until}\kern0.55em k}{k}, $$

where *k* is the gene rank. The estimated and true FDR was calculated based on 1500 randomly selected genes to make them comparable between different resampled datasets.

## Additional files

Additional file 1: Table S1. The full area under curve estimates at different group sizes for all 14 methods. Higher values represent higher gene ranking performance. The results are calculated based on 100 resampled metagenomes. The Wilcoxon-Mann–Whitney test was not evaluated at the smallest sample size (3 + 3) due to lack of samples. (DOCX 14 kb) Additional file 2: Figure S1. Ranking performance at increasing group size for the remaining methods. For each method, the receiver operating characteristics curve shows the true positive rate (y-axis) and the false positive rate (x-axis) at each position in the gene ranking list. Panels a-c show results for the Qin dataset and panels d-f show results for the Yatsunenko dataset. Group sizes of 3 + 3, 6 + 6 and 10 + 10 were included in the comparison and the effect size was fixed at a fold-change of 5. Each curve is based 100 resampled metagenomes. The methods included are the *t*-test using the square root transform (sqrtT), the *t*-test using log transform (logT), the non-transformed pooled *t*-test (tTest), Welch’s test (Welch), Wilcoxon-Mann–Whitney test (WMW), the binomial test (binomial), the non-overdispersed Poisson generalized linear model (GLM) and Fisher’s exact test (Fisher). (PDF 134 kb) Additional file 3: Figure S2. Ranking performance at increasing effect size for the main methods. The effect size (fold change) influences the ability to identify differentially abundant genes but has no considerable effect on the relative performance between the methods. The effect size varied from 3, 5 to 7 and the group sizes were fixed at 6 + 6. Panels a-c show results for the first data set and panels d-f show results for the second data set. The receiver operating characteristic curves were averaged over 100 realizations of resampled data. The included methods are edgeR, DESeq2, the overdispersed generalized linear model (OGLM), metagenomeSeq (mSeq), metastats and voom. For the results of the remaining methods see Figure S3. (PDF 131 kb) Additional File 4: Figure S3. Ranking performance at increasing effect size for the remaining methods. The effect size varies from 3, 5 to 7 and the group size is fixed at 6 + 6. Panels a-c show results for the first data set and panels d-f show results for the second data set. The receiver operating characteristic curves are averaged over 100 realizations of resampled data. The included methods are the *t*-test using the square root transform (sqrtT), the *t*-test using log transform (logT), the non-transformed pooled *t*-test (tTest), Welch’s test (Welch), Wilcoxon-Mann–Whitney test (WMW), the binomial test (binomial), the non-overdispersed Poisson generalized linear model (GLM) and Fisher’s exact test (Fisher). (PDF 135 kb) Additional file 5: Table S2. The gene ranking performance at different effect sizes for all 14 methods. Each listed value is the normalized area under curve up until a false positive rate of 0.05. Higher values represent higher gene ranking performance. The results are calculated based on 100 resampled metagenomes. The Wilcoxon-Mann–Whitney test was not evaluated at the smallest sample size (3 + 3) due to lack of samples. The full area under curve (AUC) is available in Table S3. (DOCX 13 kb) Additional file 6: Table S3. The full area under curve estimates at different effect sizes for all 14 methods. Higher values represent higher gene ranking performance. The results are calculated based on 100 resampled metagenomes. The Wilcoxon-Mann–Whitney test was not evaluated at the smallest sample size (3 + 3) due to lack of samples. (DOCX 12 kb) Additional file 7: Figure S4. Ranking performance at increasing abundance for the remaining methods. For each method, the receiver operating characteristics curve shows the true positive rate (y-axis) and the false positive rate (x-axis) at each position in the gene ranking list. Panels a-c show results for the Qin dataset and panels d-f show results for the Yatsunenko dataset. The genes were stratified into three parts based on the average number of DNA fragments, i) ≤500, ii) 500–5000 and iii) >5000 for the Qin dataset and e i) ≤10, ii) 10–50 and iii) >50 for the Yatsunenko dataset. The effect size was set to a fold-change of 5 and the group size fixed at 6 + 6 samples. Each curve is based 100 resampled metagenomes. The methods included are the *t*-test using the square root transform (sqrtT), the *t*-test using log transform (logT), the non-transformed pooled *t*-test (tTest), Welch’s test (Welch), Wilcoxon-Mann–Whitney test (WMW), the binomial test (binomial), the non-overdispersed Poisson generalized linear model (GLM) and Fisher’s exact test (Fisher). (PDF 133 kb) Additional file 8: Table S4. The full area under curve estimates at different gene abundances for all 14 methods. Higher values represent higher gene ranking performance. The results are calculated based on 100 resampled metagenomes. The Wilcoxon-Mann–Whitney test was not evaluated at the smallest sample size (3 + 3) due to lack of samples. (DOCX 12 kb) Additional file 9: Figure S5. Accuracy of effect estimates. Panels a-c show results for the Qin dataset and panels d-f show results for the Yatsunenko dataset. The non-overdispersed Poisson generalized linear model (GLM) has identical effect estimates to the OGLM and is not shown in the figure. Group sizes of 3 + 3, 6 + 6 and 10 + 10 were included in the comparison and the effect size was fixed at a fold-change of 5. Each plot is based 100 resampled metagenomes. (PDF 108 kb) Additional file 10: Figure S6. The *p*-value distributions for the Qin dataset for the remaining methods. For the quantile-quantile-plots, each grey line represent a resampled metagenome, the solid black line represents the average value and the dotted line the line with slope one corresponding to a uniform *p*-value distribution. The *p*-value distributions were created based on 100 resampled metagenomes with no added effect and a group size of 6 + 6. (PDF 2375 kb) Additional file 11: Figure S7. The *p*-value distributions for the Yatsunenko dataset for the remaining methods. For the quantile-quantile-plots, each grey line represent a resampled metagenome, the solid black line represents the average value and the dotted line the line with slope one corresponding to a uniform *p*-value distribution. The *p*-value distributions were created based on 100 resampled metagenomes with no added effect and a group size of 6 + 6. (PDF 3526 kb) Additional file 12: Figure S8. The ability to control the false discovery rate for the remaining methods. The figure shows boxplots of the number of true positives (panel a, d), the number of false positives (panel b, e) and achieved true FDR (panel c, f) at a cutoff of 0.05 estimated FDR. Panels a-c show results for the Qin dataset and panels d-f show results for the Yatsunenko dataset. The group sizes were set to 6 + 6 and the effect size to 5. The results were based on 100 resampled metagenomes. The included methods are the *t*-test using the square root transform (sqrtT), the *t*-test using log transform (logT), the non-transformed pooled *t*-test (tTest), Welch’s test (Welch), Wilcoxon-Mann–Whitney test (WMW), the binomial test (binomial), the non-overdispersed Poisson generalized linear model (GLM) and Fisher’s exact test (Fisher). (PDF 117 kb) Additional file 13: Table S5. Summary of results from the FDR analysis. The median value for the number of true positives detected, the number of false positives detected and achieved true FDR at a cutoff of 0.05 estimated FDR for both data sets. The group sizes were set to 6 + 6 and the effect size to 5. The results were based on 100 resampled metagenomes. (DOCX 15 kb) Additional file 14: Figure S9. The abundance of zeroes averaged over resampled data sets. There is a large difference in the number of zeros for the low abundant genes but small differences for the more highly abundant genes. The cutoffs in average number of DNA fragments were the same as used in previous results, i) ≤500, ii) 500–5000 and iii) >5000 for the Qin dataset and e i) ≤10, ii) 10–50 and iii) >50 for the Yatsunenko dataset. The group size was fixed to 6 + 6. Error bars indicate the 95 % confidence interval for the mean between resampled data sets. (PDF 19 kb) Additional file 15: Table S6. Area under curve estimates on unfiltered data for all 14 methods. Higher values represent higher gene ranking performance. The results are calculated based on 100 resampled metagenomes. The Group size was 6 + 6 and the effect fixed to 5. (DOCX 12 kb)

## References

[1] Handelsman J, Rondon MR, Brady SF, Clardy J, Goodman RM (1998). Molecular biological access to the chemistry of unknown soil microbes: a new frontier for natural products. Chem Biol.

[2] Rondon MR, August PR, Bettermann AD, Brady SF, Grossman TH, Liles MR (2000). Cloning the soil metagenome: A strategy for accessing the genetic and functional diversity of uncultured microorganisms. Appl Environ Microbiol.

[3] Schloss PD, Handelsman J (2005). Metagenomics for studying unculturable microorganisms: cutting the Gordian knot. Genome Biol.

[4] Qin J, Li Y, Cai Z, Li S, Zhu J, Zhang F (2012). A metagenome-wide association study of gut microbiota in type 2 diabetes. Nature.

[5] Yatsunenko T, Rey FE, Manary MJ, Trehan I, Dominguez-Bello MG, Contreras M (2012). Human gut microbiome viewed across age and geography. Nature.

[6] Karlsson FH, Tremaroli V, Nookaew I, Bergström G, Behre CJ, Fagerberg B (2013). Gut metagenome in European women with normal, impaired and diabetic glucose control. Nature.

[7] Delmont TO, Robe P, Cecillon S, Clark IM, Constancias F, Simonet P (2011). Accessing the soil metagenome for studies of microbial diversity. Appl Environ Microbiol.

[8] Kelley ST, Gilbert JA (2013). Studying the microbiology of the indoor environment. Genome Biol.

[9] Ferreira AJS, Siam R, Setubal JC, Moustafa A, Sayed A, Chambergo FS, et al. Core microbial functional activities in ocean environments revealed by global metagenomic profiling analyses. PLoS One. 2014;9. 10.1371/journal.pone.0097338 PMC4055538 24921648

[10] Wooley JC, Godzik A, Friedberg I (2010). A primer on metagenomics. PLoS Comput Biol.

[11] Wooley JC, Ye Y (2009). Metagenomics: Facts and artifacts, and computational challenges. J Comput Sci Technol.

[12] Knight R, Jansson J, Field D, Fierer N, Desai N, Fuhrman JA (2012). Unlocking the potential of metagenomics through replicated experimental design. Nat Biotechnol.

[13] Chafee M, Maignien L, Simmons SL (2015). The effects of variable sample biomass on comparative metagenomics. Environ Microbiol.

[14] Brooks JP, Edwards DJ, Harwich MD, Rivera MC, Fettweis JM, Serrano MG (2015). The truth about metagenomics: quantifying and counteracting bias in 16S rRNA studies. BMC Microbiol.

[15] Rodriguez-Brito B, Rohwer F, Edwards RA (2006). An application of statistics to comparative metagenomics. BMC Bioinformatics.

[16] Markowitz VM, Ivanova NN, Szeto E, Palaniappan K, Chu K, Dalevi D, et al. IMG/M: A data management and analysis system for metagenomes. Nucleic Acids Res. 2008;36 Suppl 1:D534–8. 10.1093/nar/gkm869 PMC2238950 17932063

[17] Markowitz VM, Chen I-M A, Chu K, Szeto E, Palaniappan K, Grechkin Y (2012). IMG/M: the integrated metagenome data management and comparative analysis system. Nucleic Acids Res.

[18] Kristiansson E, Hugenholtz P, Dalevi D (2009). ShotgunFunctionalizeR: an R-package for functional comparison of metagenomes. Bioinformatics.

[19] White JR, Nagarajan N, Pop M (2009). Statistical methods for detecting differentially abundant features in clinical metagenomic samples. PLoS Comput Biol.

[20] Parks DH, Tyson GW, Hugenholtz P, Beiko RG (2014). STAMP: statistical analysis of taxonomic and functional profiles. Bioinformatics.

[21] Parks DH, Beiko RG (2010). Identifying biologically relevant differences between metagenomic communities. Bioinformatics.

[22] Segata N, Izard J, Waldron L, Gevers D, Miropolsky L, Garrett WS (2011). Metagenomic biomarker discovery and explanation. Genome Biol.

[23] Sanli K, Karlsson FH, Nookaew I, Nielsen J (2013). FANTOM: Functional and taxonomic analysis of metagenomes. BMC Bioinformatics.

[24] Paulson JN, Stine OC, Bravo HC, Pop M (2013). Differential abundance analysis for microbial marker-gene surveys. Nat Methods.

[25] Smyth GK (2004). Linear Models and Empirical Bayes Methods for Assessing Differential Expression in Microarray Experiments. Stat Appl Genet Mol Biol.

[26] Robinson MD, McCarthy DJ, Smyth GK (2010). edgeR: a Bioconductor package for differential expression analysis of digital gene expression data. Bioinformatics.

[27] Ross EM, Moate PJ, Marett L, Cocks BG, Hayes BJ (2013). Investigating the effect of two methane-mitigating diets on the rumen microbiome using massively parallel sequencing. J Dairy Sci.

[28] Love MI, Huber W, Anders S (2014). Moderated estimation of fold change and dispersion for RNA-Seq data with DESeq2. Genome Biol.

[29] Dugat-Bony E, Straub C, Teissandier A, Onésime D, Loux V, Monnet C (2015). Overview of a Surface-Ripened Cheese Community Functioning by Meta-Omics Analyses. PLoS ONE.

[30] Law CW, Chen Y, Shi W, Smyth GK (2014). Voom: precision weights unlock linear model analysis tools for RNA-seq read counts. Genome Biol.

[31] Bragina A, Oberauner-Wappis L, Zachow C, Halwachs B, Thallinger GG, Müller H, et al. The Sphagnum microbiome supports bog ecosystem functioning under extreme conditions. Mol Ecol. 2014;4498–4510. 10.1111/mec.12885 25113243

[32] Nookaew I, Papini M, Pornputtapong N, Scalcinati G, Fagerberg L, Uhlén M (2012). A comprehensive comparison of RNA-Seq-based transcriptome analysis from reads to differential gene expression and cross-comparison with microarrays: a case study in Saccharomyces cerevisiae. Nucleic Acids Res.

[33] Soneson C, Delorenzi M (2013). A comparison of methods for differential expression analysis of RNA-seq data. BMC Bioinformatics.

[34] McCullagh P, Nelder JA (1989). Generalized Linear Models.

[35] Anscombe FJ (1948). The Transformation of Poisson. Binomial and Negative-Binomial Data. Biometrika.

[36] Lehmann EL, D’Abrera HJM (2006). Nonparametrics: Statistical Methods Based on Ranks.

[37] Casella G, Berger RL (2002). Statistical Inference.

[38] McMurdie PJ, Holmes S (2014). Waste not, want not: why rarefying microbiome data is inadmissible. PLoS Comput Biol.

[39] Sjögren A, Kristiansson E, Rudemo M, Nerman O (2007). Weighted analysis of general microarray experiments. BMC Bioinformatics.

[40] Liu R, Holik AZ, Su S, Jansz N, Chen K, Leong HS (2015). Why weight? Modelling sample and observational level variability improves power in RNA-seq analyses. Nucleic Acids Res.

[41] Benjamini Y, Hochberg Y (1995). Controlling the false discovery rate: a practical and powerful approach to multiple testing. J R Stat Soc Ser B.

[42] Storey JD, Tibshirani R (2003). Statistical significance for genomewide studies. Proc Natl Acad Sci U S A.

[43] Karlsson FH, Nookaew I, Nielsen J (2014). Metagenomic Data Utilization and Analysis (MEDUSA) and Construction of a Global Gut Microbial Gene Catalogue. PLoS Comput Biol.

[44] Alneberg J, Bjarnason BS, de Bruijn I, Schirmer M, Quick J, Ijaz UZ (2014). Binning metagenomic contigs by coverage and composition. Nat Methods.

[45] Schwartzman A, Lin X (2011). The effect of correlation in false discovery rate estimation. Biometrika.

[46] Beszteri B, Temperton B, Frickenhaus S, Giovannoni SJ (2010). Average genome size: a potential source of bias in comparative metagenomics. ISME J.

[47] Sohn MB, Du R, An L (2015). A robust approach for identifying differentially abundant features in metagenomic samples. Bioinformatics.

[48] Frank JA, Sorensen SJ (2011). Quantitative Metagenomic Analyses Based on Average Genome Size Normalization. Appl Environ Microbiol.

[49] Robinson MD, Smyth GK (2007). Moderated statistical tests for assessing differences in tag abundance. Bioinformatics.

[50] McCarthy DJ, Chen Y, Smyth GK (2012). Differential expression analysis of multifactor RNA-Seq experiments with respect to biological variation. Nucleic Acids Res.

[51] R Core Team. R: A Language and Environment for Statistical Computing. Vienna, Austria; 2014. http://www.R-project.org/

[52] Hollander M, Wolfe DA. Nonparametric Statistical Methods. New York: John Wiley & Sons; 1999.

[53] Robinson MD, Oshlack A (2010). A scaling normalization method for differential expression analysis of RNA-seq data. Genome Biol.

[54] Anders S, Huber W (2010). Differential expression analysis for sequence count data. Genome Biol.

[55] Qin J, Li R, Raes J, Arumugam M, Burgdorf KS, Manichanh C (2010). A human gut microbial gene catalogue established by metagenomic sequencing. Nature.

[56] Haft DH (2003). The TIGRFAMs database of protein families. Nucleic Acids Res.

[57] Eddy SR (2011). Accelerated Profile HMM Searches. PLoS Comput Biol.

[58] Boulund F, Sjögren A, Kristiansson E (2015). Tentacle: distributed quantification of genes in metagenomes. Gigascience.

[59] Kent WJ (2002). BLAT---The BLAST-Like Alignment Tool. Genome Res.

[60] Meyer F, Paarmann D, D’Souza M, Olson R, Glass EM, Kubal M (2008). The metagenomics RAST server - a public resource for the automatic phylogenetic and functional analysis of metagenomes. BMC Bioinformatics.

[61] Fawcett T (2006). An introduction to ROC analysis. Pattern Recognit Lett.

