# Supplementary material for: Statistical evaluation of methods for identification of differentially abundant genes in comparative metagenomics
Source: BMC Genomics. 2016 Jan 25;17:78. doi: 10.1186/s12864-016-2386-y (PMC4727335; doi:10.1186/s12864-016-2386-y)
Supplement: Additional file 1: Table S1. — The full area under curve estimates at different group sizes for all 14 methods. Higher values represent higher gene ranking performance. The results are calculated based on 100 resampled metagenomes. The Wilcoxon-Mann–Whitney test was not evaluated at the smallest sample size (3 + 3) due to lack of samples. (DOCX 14 kb) [file 12864_2016_2386_MOESM1_ESM.docx]

**Table S1. The full area under curve estimates at different group sizes for all 14 methods.**

| **AUC** | **Data set 1: (Qin 2010)** | | | **Data set 2: (Yatsunenko 2012)** | | |
| --- | --- | --- | --- | --- | --- | --- |
| **Group Size** | **3+3** | **6+6** | **10+10** | **3+3** | **6+6** | **10+10** |
| **edgeR** | 0.91 | 0.95 | 0.97 | 0.91 | 0.97 | 0.99 |
| **DESeq2** | 0.90 | 0.94 | 0.96 | 0.89 | 0.96 | 0.99 |
| **OGLM** | 0.93 | 0.96 | 0.98 | 0.87 | 0.95 | 0.98 |
| **MetagenomeSeq** | 0.90 | 0.96 | 0.98 | 0.85 | 0.94 | 0.98 |
| **Metastats** | 0.84 | 0.90 | 0.92 | 0.81 | 0.92 | 0.97 |
| **voom** | 0.87 | 0.90 | 0.92 | 0.85 | 0.92 | 0.97 |
| **Sqrt t-test** | 0.89 | 0.93 | 0.96 | 0.86 | 0.94 | 0.98 |
| **Log t-test** | 0.88 | 0.90 | 0.92 | 0.85 | 0.93 | 0.97 |
| **t-test** | 0.90 | 0.95 | 0.97 | 0.86 | 0.94 | 0.98 |
| **Welch t-test** | 0.89 | 0.94 | 0.97 | 0.85 | 0.94 | 0.98 |
| **WMW** | ----- | 0.92 | 0.94 | ----- | 0.93 | 0.97 |
| **binomial** | 0.87 | 0.91 | 0.93 | 0.84 | 0.91 | 0.96 |
| **GLM** | 0.87 | 0.90 | 0.92 | 0.84 | 0.92 | 0.96 |
| **Fisher’s exact test** | 0.87 | 0.90 | 0.92 | 0.84 | 0.91 | 0.96 |

Higher values represent higher gene ranking performance. The results are calculated based on 100 resampled metagenomes. The Wilcoxon-Mann-Whitney test was not evaluated at the smallest sample size (3+3) due to lack of samples.
