# Supplementary figures and images for: Statistical evaluation of methods for identification of differentially abundant genes in comparative metagenomics
Source: BMC Genomics. 2016 Jan 25;17:78. doi: 10.1186/s12864-016-2386-y (PMC4727335; doi:10.1186/s12864-016-2386-y)

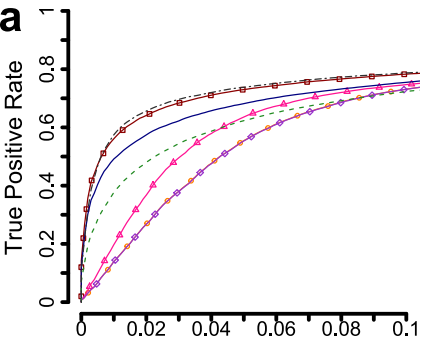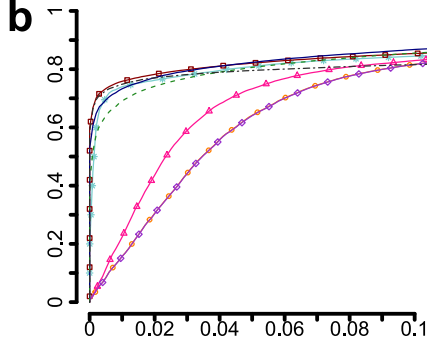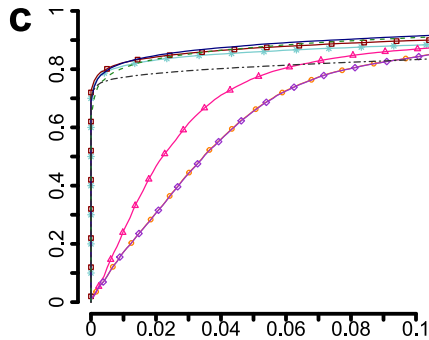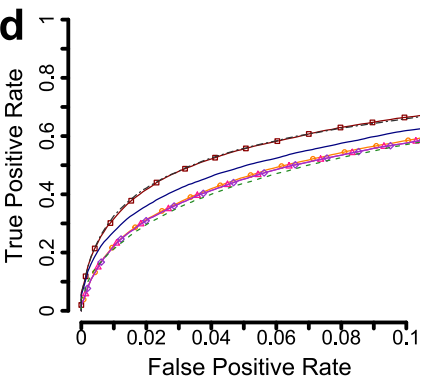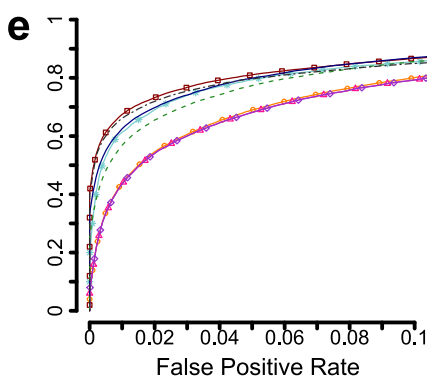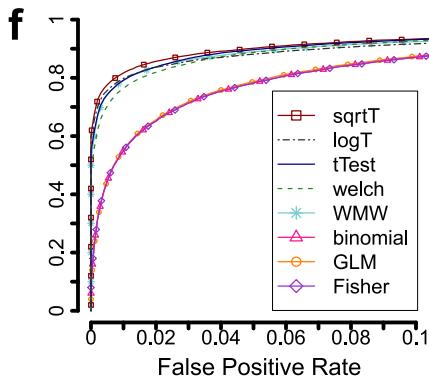

Increasing Group Sizes

Supplement: Additional file 2: Figure S1. — Ranking performance at increasing group size for the remaining methods. For each method, the receiver operating characteristics curve shows the true positive rate (y-axis) and the false positive rate (x-axis) at each position in the gene ranking list. Panels a-c show results for the Qin dataset and panels d-f show results for the Yatsunenko dataset. Group sizes of 3 + 3, 6 + 6 and 10 + 10 were included in the comparison and the effect size was fixed at a fold-change of 5. Each curve is based 100 resampled metagenomes. The methods included are the t-test using the square root transform (sqrtT), the t-test using log transform (logT), the non-transformed pooled t-test (tTest), Welch’s test (Welch), Wilcoxon-Mann–Whitney test (WMW), the binomial test (binomial), the non-overdispersed Poisson generalized linear model (GLM) and Fisher’s exact test (Fisher). (PDF 134 kb) [file 12864_2016_2386_MOESM2_ESM.pdf]

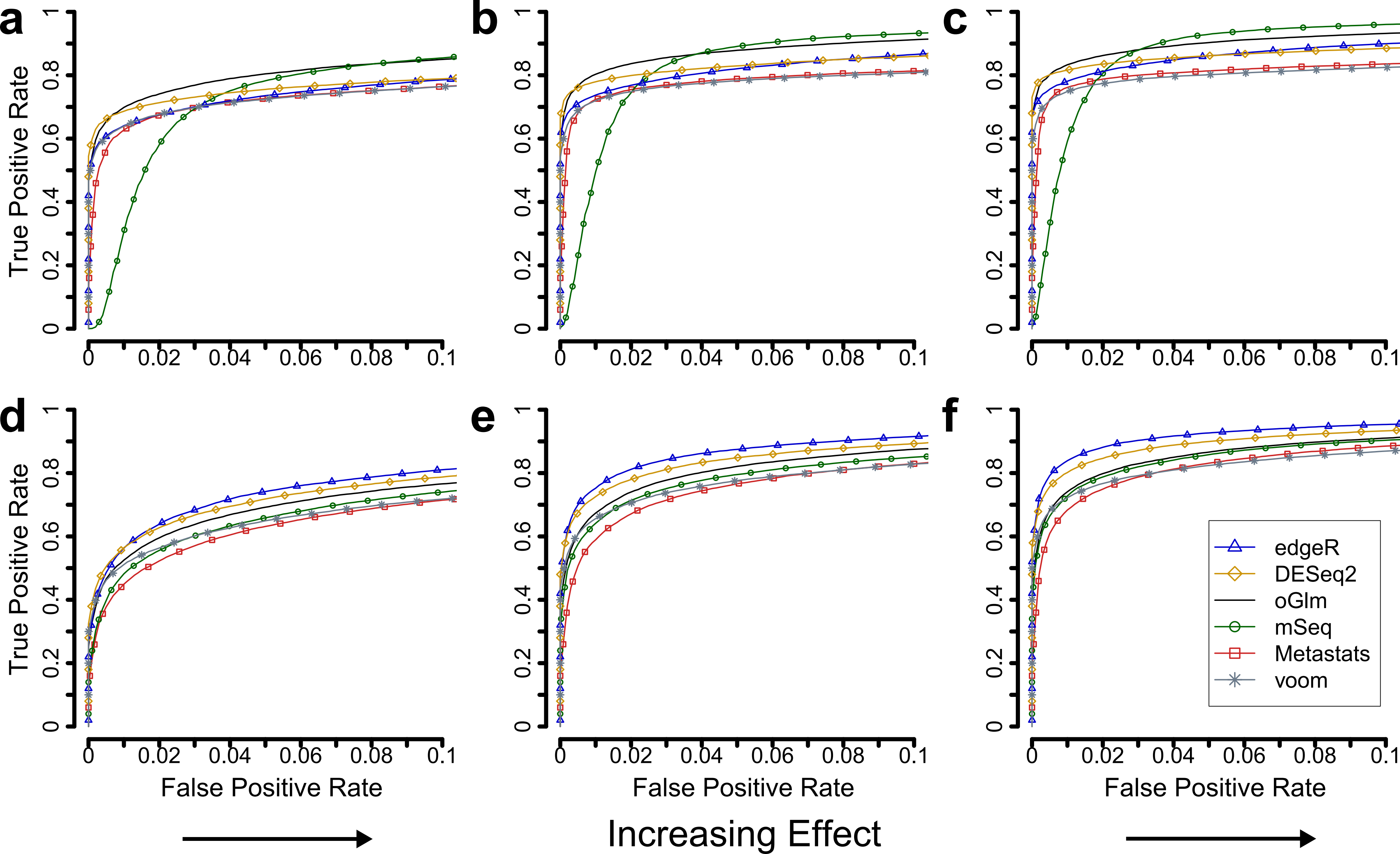

Supplement: Additional file 3: Figure S2. — Ranking performance at increasing effect size for the main methods. The effect size (fold change) influences the ability to identify differentially abundant genes but has no considerable effect on the relative performance between the methods. The effect size varied from 3, 5 to 7 and the group sizes were fixed at 6 + 6. Panels a-c show results for the first data set and panels d-f show results for the second data set. The receiver operating characteristic curves were averaged over 100 realizations of resampled data. The included methods are edgeR, DESeq2, the overdispersed generalized linear model (OGLM), metagenomeSeq (mSeq), metastats and voom. For the results of the remaining methods see Figure S3. (PDF 131 kb) [file 12864_2016_2386_MOESM3_ESM.pdf]

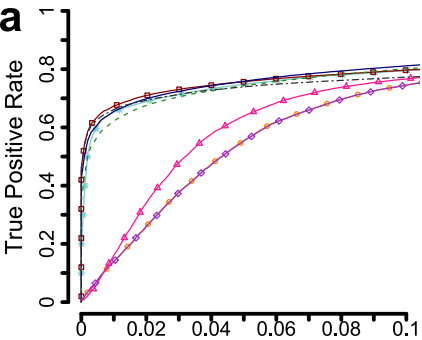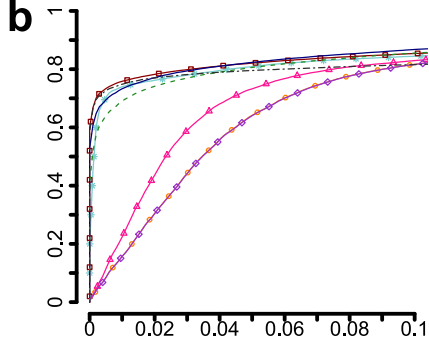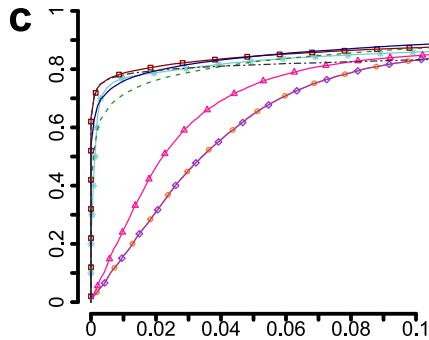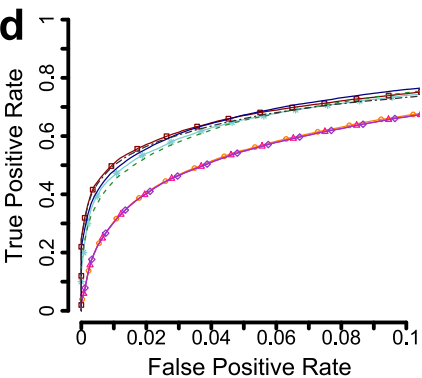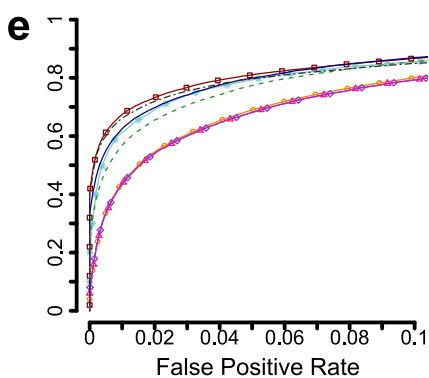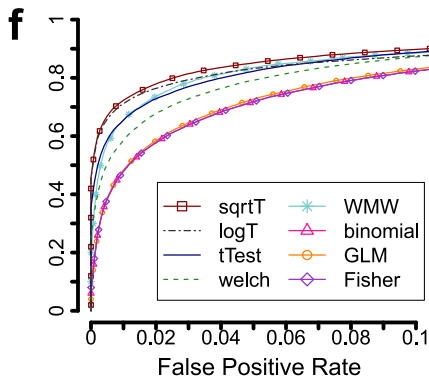

Increasing Effect

Supplement: Additional File 4: Figure S3. — Ranking performance at increasing effect size for the remaining methods. The effect size varies from 3, 5 to 7 and the group size is fixed at 6 + 6. Panels a-c show results for the first data set and panels d-f show results for the second data set. The receiver operating characteristic curves are averaged over 100 realizations of resampled data. The included methods are the t-test using the square root transform (sqrtT), the t-test using log transform (logT), the non-transformed pooled t-test (tTest), Welch’s test (Welch), Wilcoxon-Mann–Whitney test (WMW), the binomial test (binomial), the non-overdispersed Poisson generalized linear model (GLM) and Fisher’s exact test (Fisher). (PDF 135 kb) [file 12864_2016_2386_MOESM4_ESM.pdf]

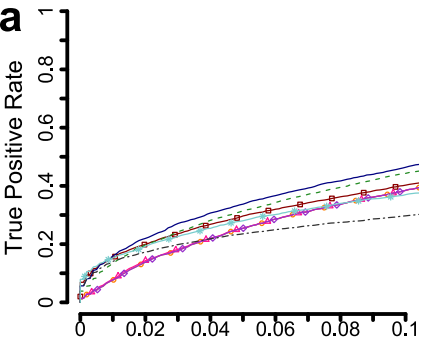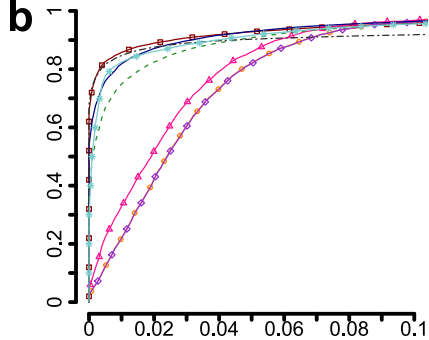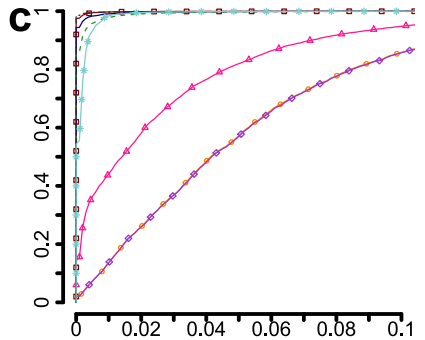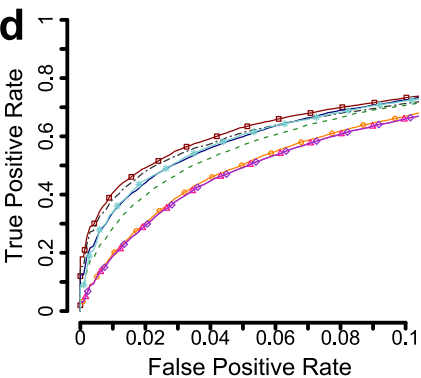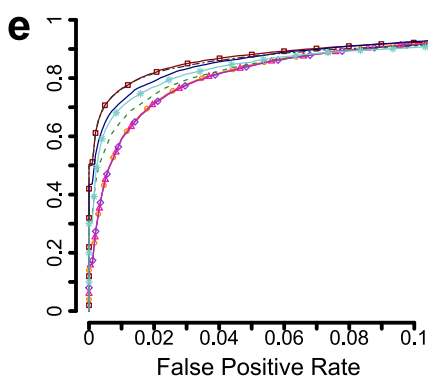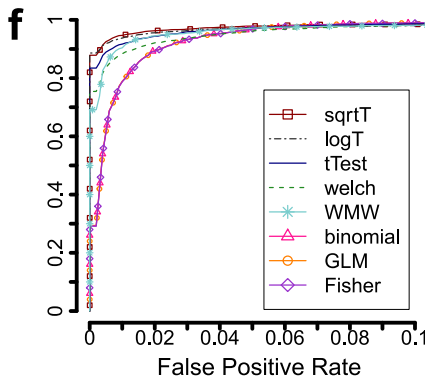

Increasing Abundance

Supplement: Additional file 7: Figure S4. — Ranking performance at increasing abundance for the remaining methods. For each method, the receiver operating characteristics curve shows the true positive rate (y-axis) and the false positive rate (x-axis) at each position in the gene ranking list. Panels a-c show results for the Qin dataset and panels d-f show results for the Yatsunenko dataset. The genes were stratified into three parts based on the average number of DNA fragments, i) ≤500, ii) 500–5000 and iii) >5000 for the Qin dataset and e i) ≤10, ii) 10–50 and iii) >50 for the Yatsunenko dataset. The effect size was set to a fold-change of 5 and the group size fixed at 6 + 6 samples. Each curve is based 100 resampled metagenomes. The methods included are the t-test using the square root transform (sqrtT), the t-test using log transform (logT), the non-transformed pooled t-test (tTest), Welch’s test (Welch), Wilcoxon-Mann–Whitney test (WMW), the binomial test (binomial), the non-overdispersed Poisson generalized linear model (GLM) and Fisher’s exact test (Fisher). (PDF 133 kb) [file 12864_2016_2386_MOESM7_ESM.pdf]

3 + 3 Qin

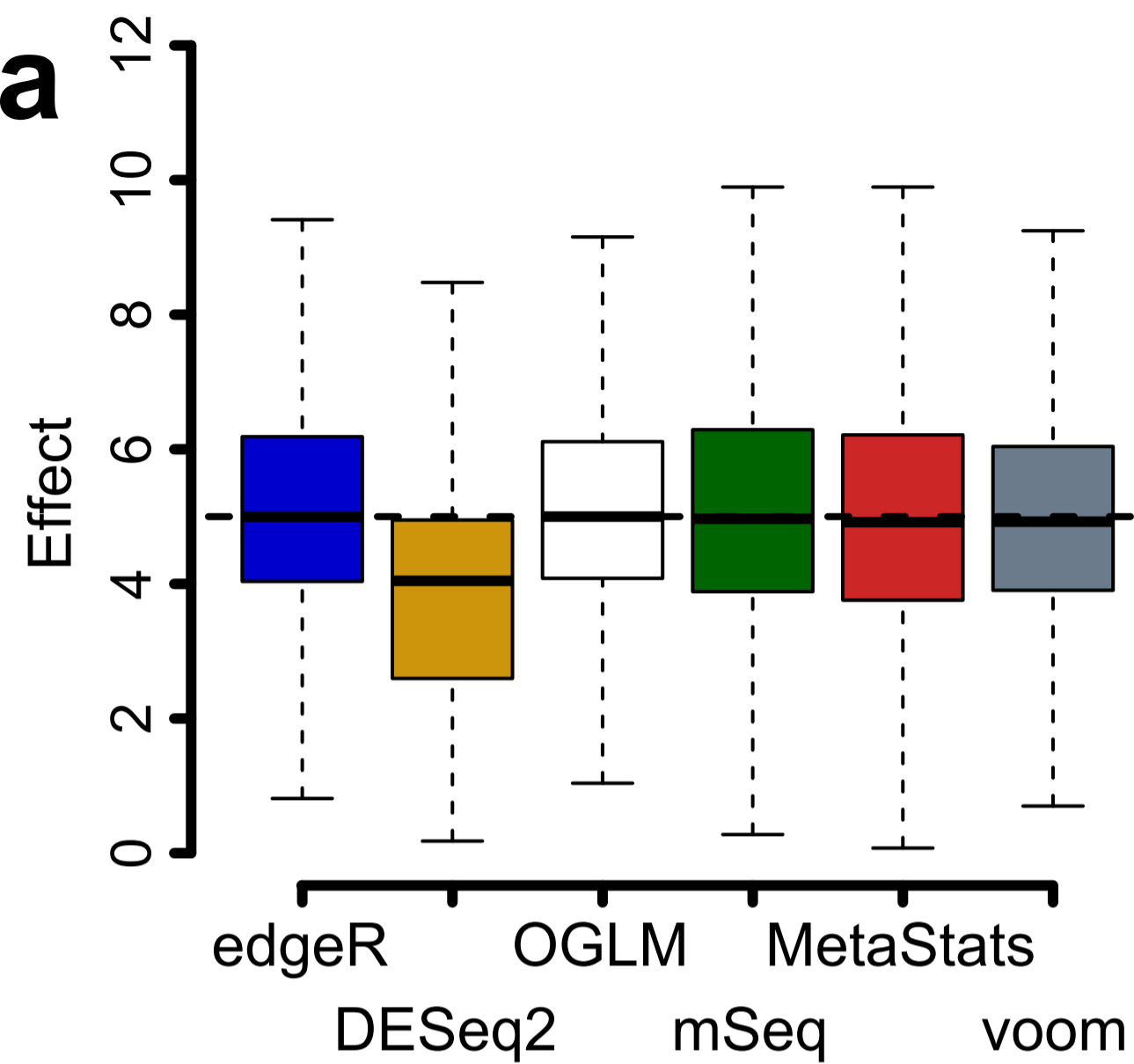

6 + 6 Qin

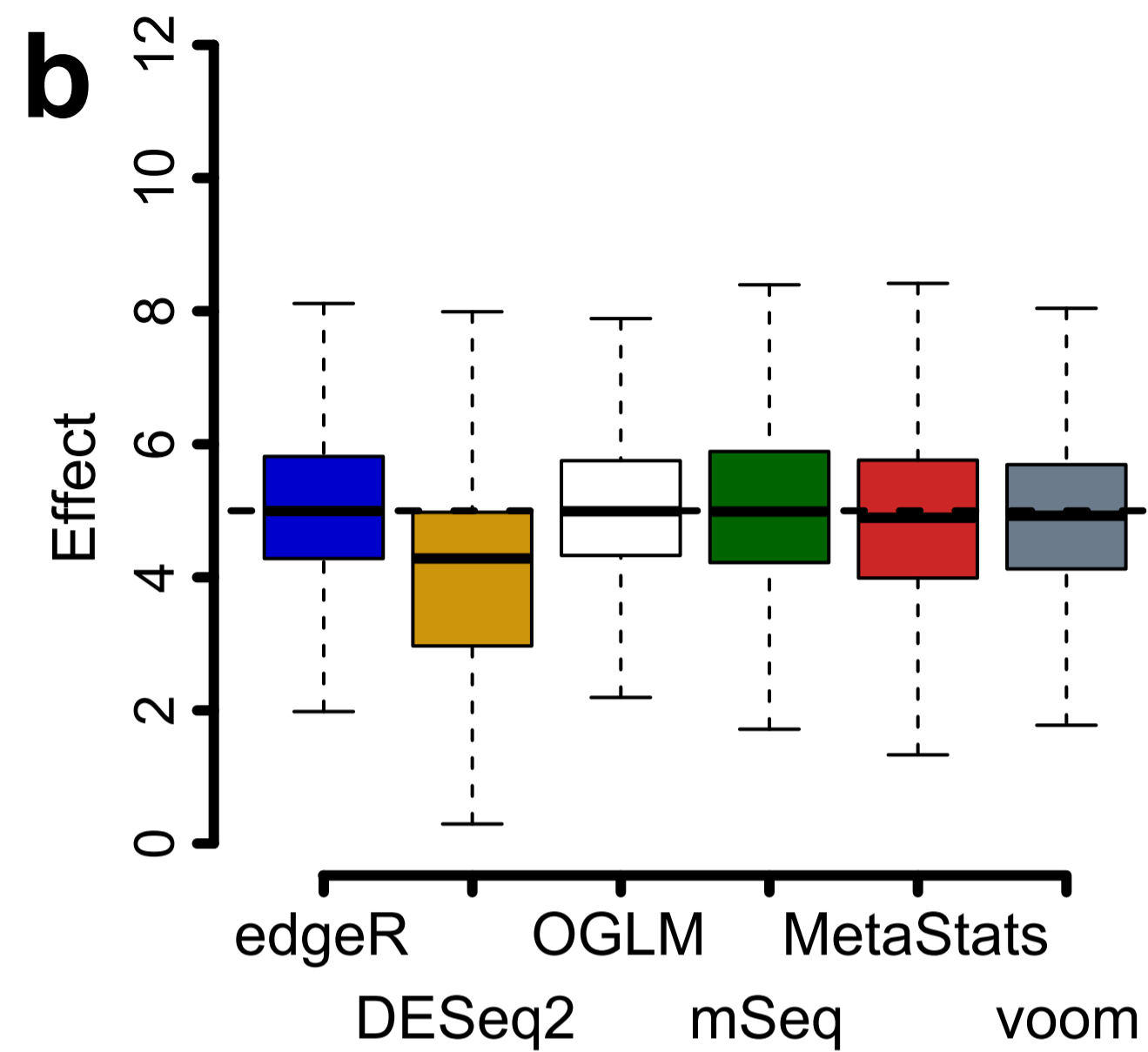

10 + 10 Qin

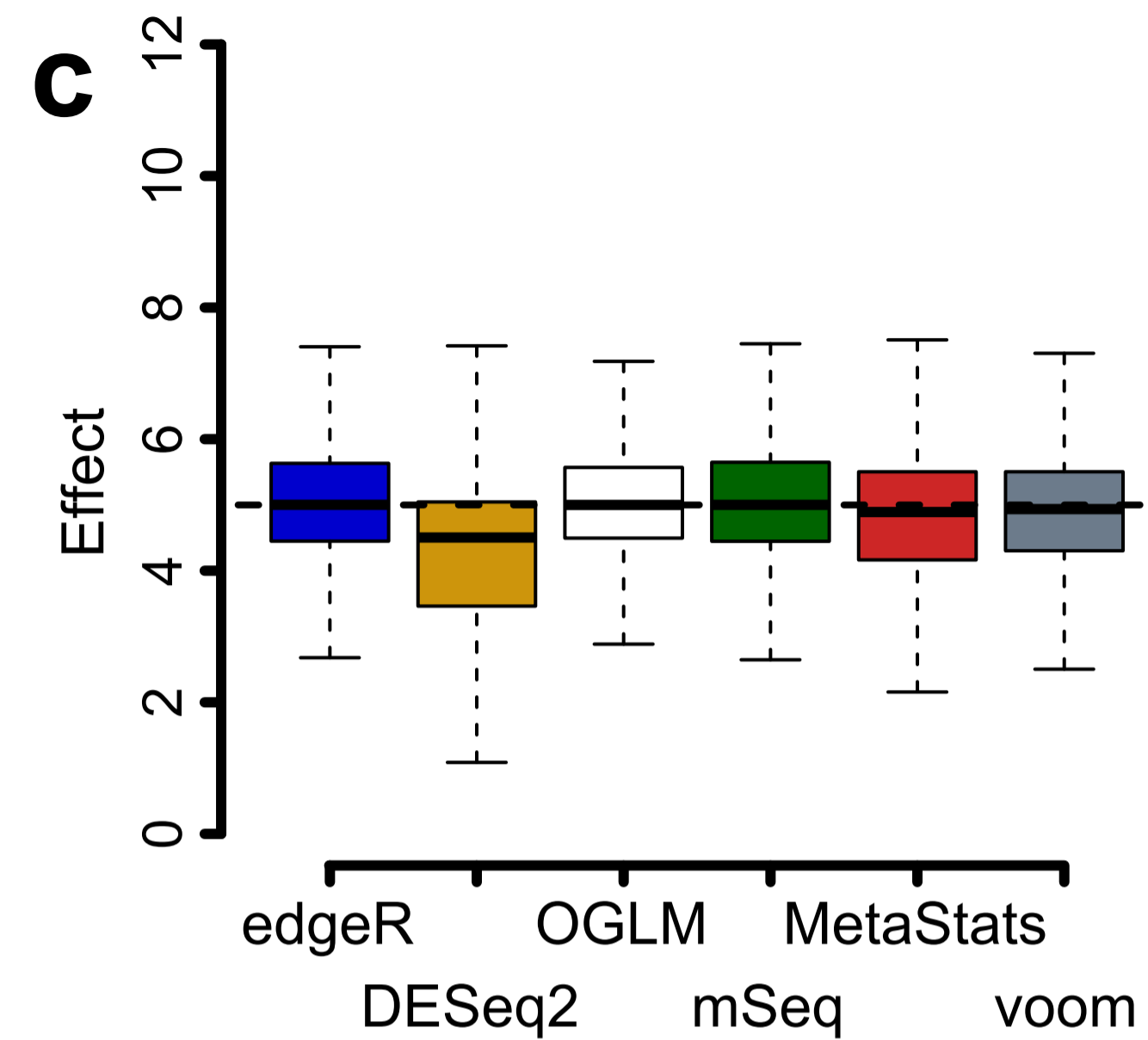

3 + 3 Yatsunenkeno

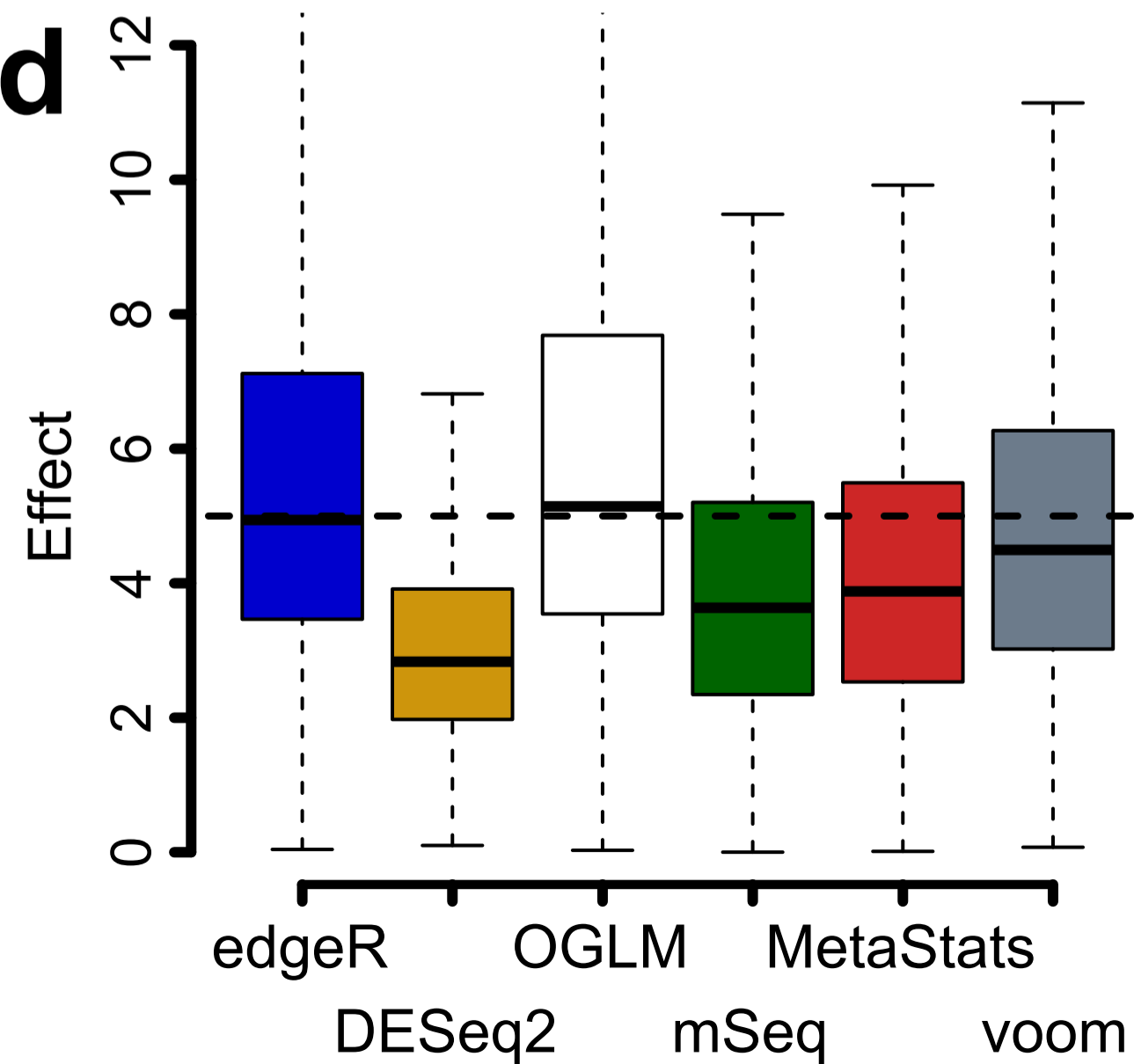

6 + 6 Yatsunenkeno

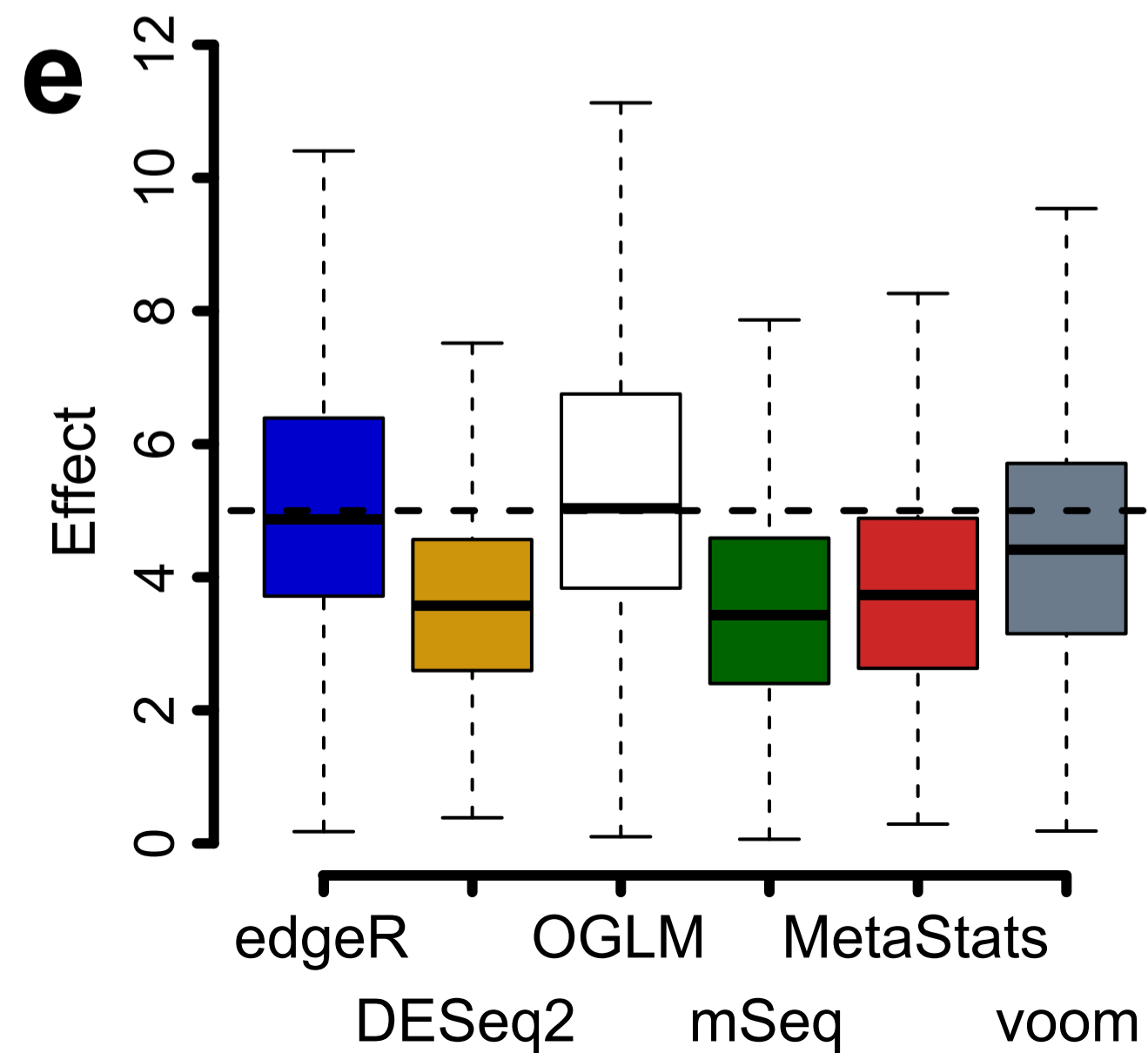

10 + 10 Yatsunenkeno

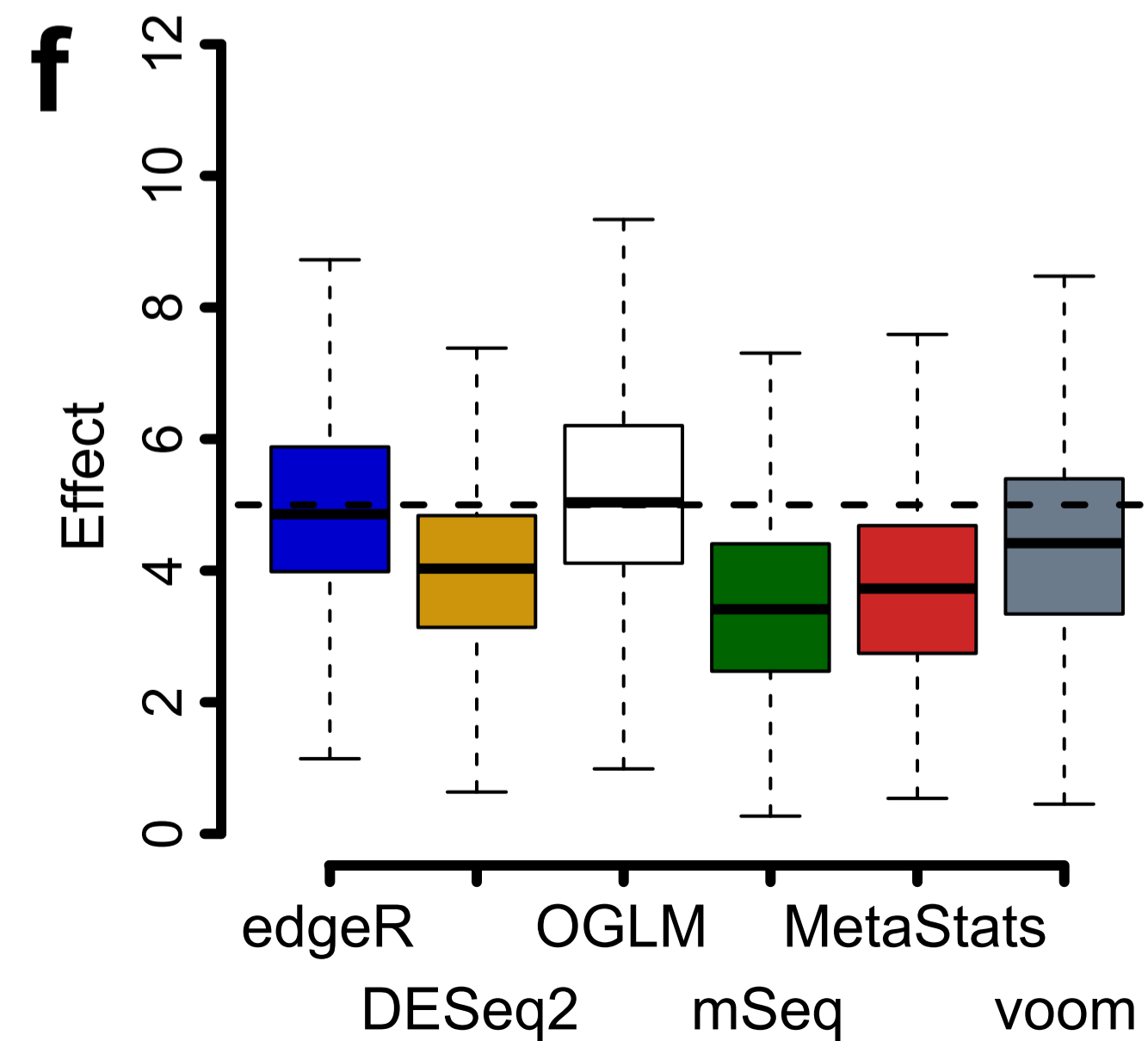

Supplement: Additional file 9: Figure S5. — Accuracy of effect estimates. Panels a-c show results for the Qin dataset and panels d-f show results for the Yatsunenko dataset. The non-overdispersed Poisson generalized linear model (GLM) has identical effect estimates to the OGLM and is not shown in the figure. Group sizes of 3 + 3, 6 + 6 and 10 + 10 were included in the comparison and the effect size was fixed at a fold-change of 5. Each plot is based 100 resampled metagenomes. (PDF 108 kb) [file 12864_2016_2386_MOESM9_ESM.pdf]

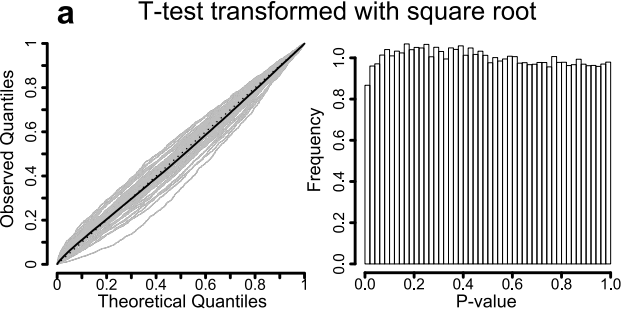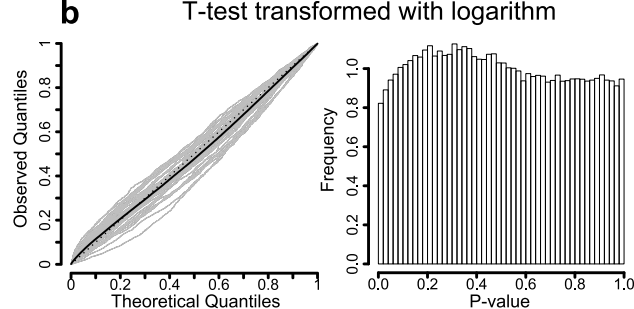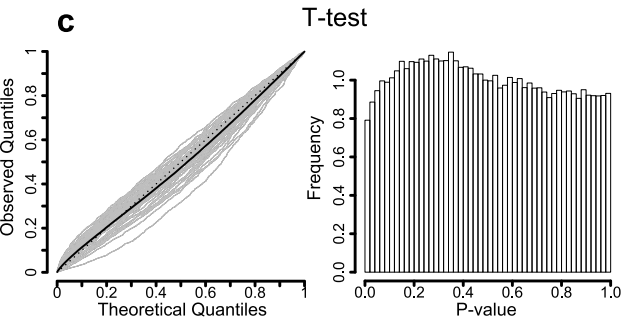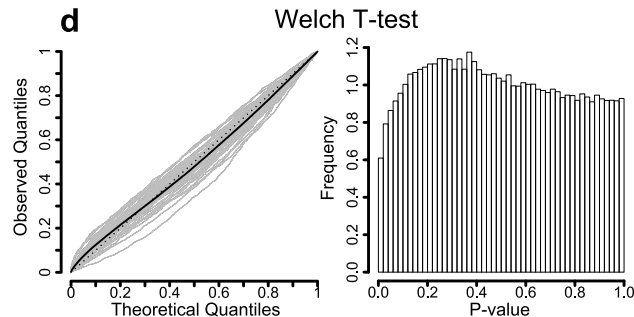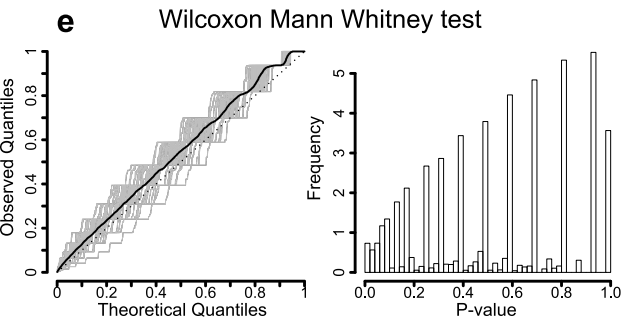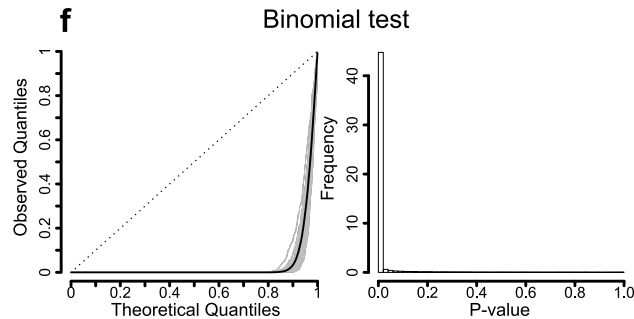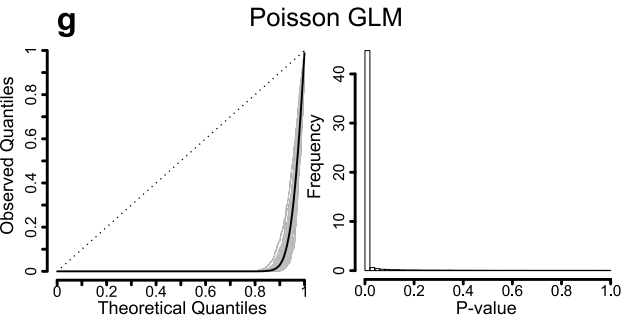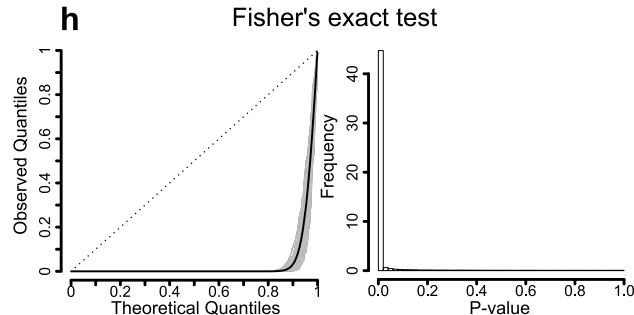

Supplement: Additional file 10: Figure S6. — The p-value distributions for the Qin dataset for the remaining methods. For the quantile-quantile-plots, each grey line represent a resampled metagenome, the solid black line represents the average value and the dotted line the line with slope one corresponding to a uniform p-value distribution. The p-value distributions were created based on 100 resampled metagenomes with no added effect and a group size of 6 + 6. (PDF 2375 kb) [file 12864_2016_2386_MOESM10_ESM.pdf]

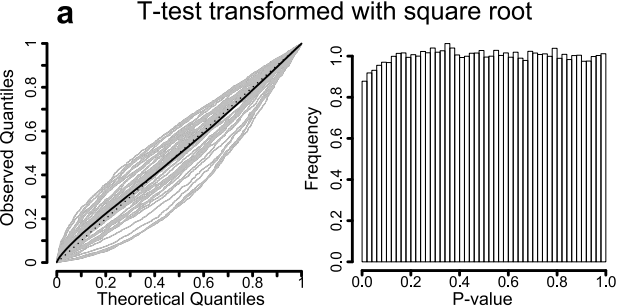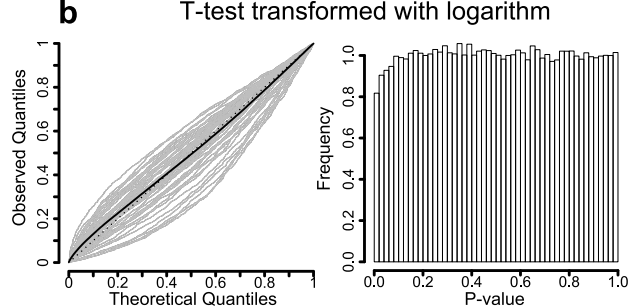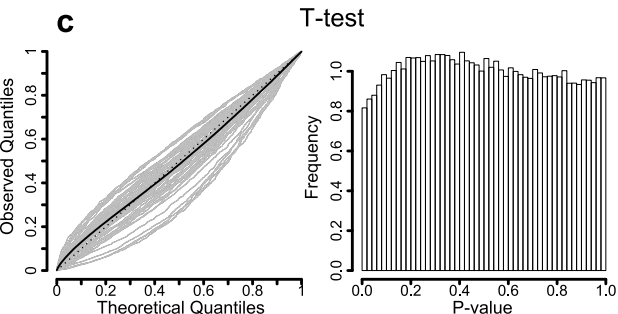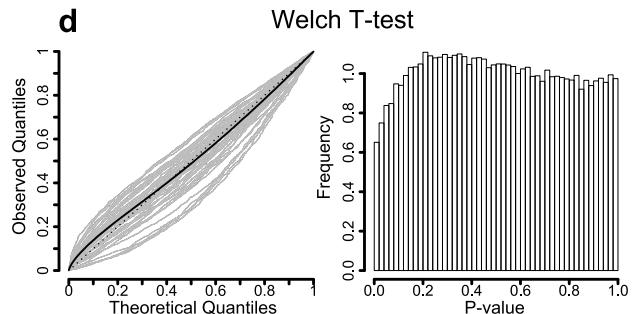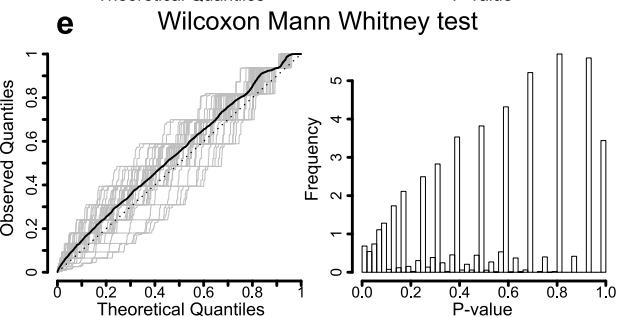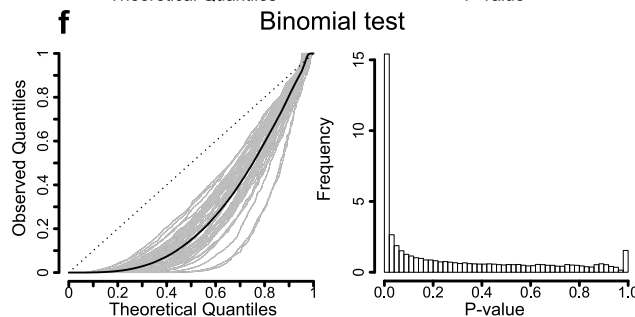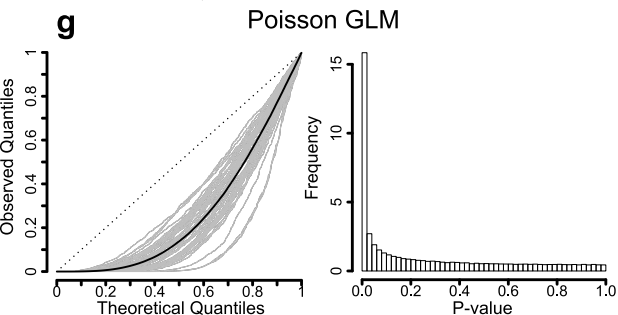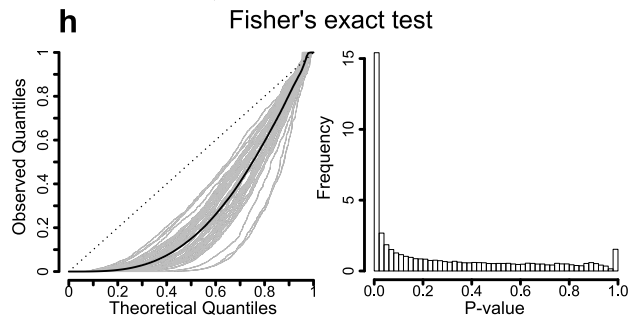

Supplement: Additional file 11: Figure S7. — The p-value distributions for the Yatsunenko dataset for the remaining methods. For the quantile-quantile-plots, each grey line represent a resampled metagenome, the solid black line represents the average value and the dotted line the line with slope one corresponding to a uniform p-value distribution. The p-value distributions were created based on 100 resampled metagenomes with no added effect and a group size of 6 + 6. (PDF 3526 kb) [file 12864_2016_2386_MOESM11_ESM.pdf]

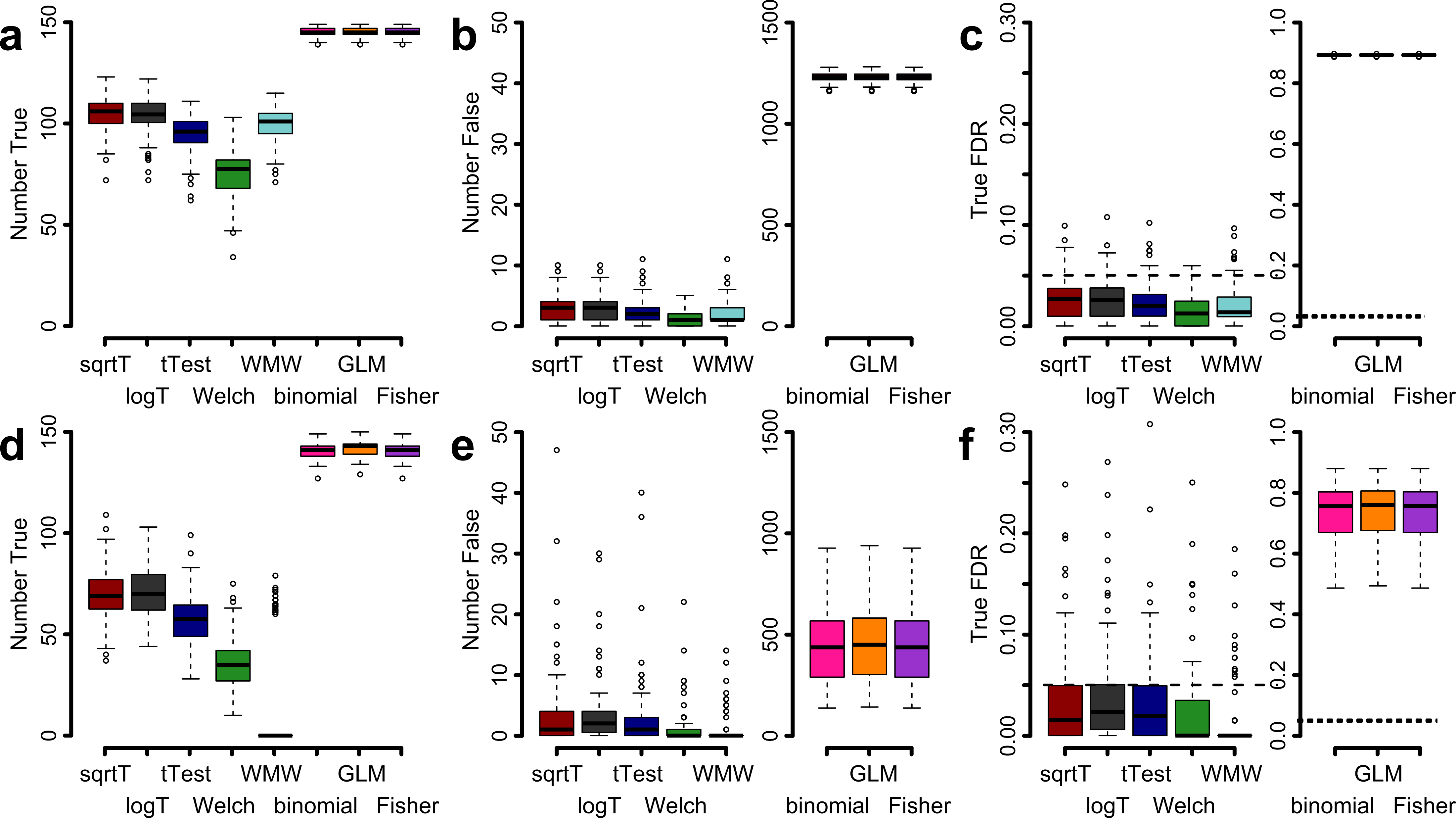

Supplement: Additional file 12: Figure S8. — The ability to control the false discovery rate for the remaining methods. The figure shows boxplots of the number of true positives (panel a, d), the number of false positives (panel b, e) and achieved true FDR (panel c, f) at a cutoff of 0.05 estimated FDR. Panels a-c show results for the Qin dataset and panels d-f show results for the Yatsunenko dataset. The group sizes were set to 6 + 6 and the effect size to 5. The results were based on 100 resampled metagenomes. The included methods are the t-test using the square root transform (sqrtT), the t-test using log transform (logT), the non-transformed pooled t-test (tTest), Welch’s test (Welch), Wilcoxon-Mann–Whitney test (WMW), the binomial test (binomial), the non-overdispersed Poisson generalized linear model (GLM) and Fisher’s exact test (Fisher). (PDF 117 kb) [file 12864_2016_2386_MOESM12_ESM.pdf]

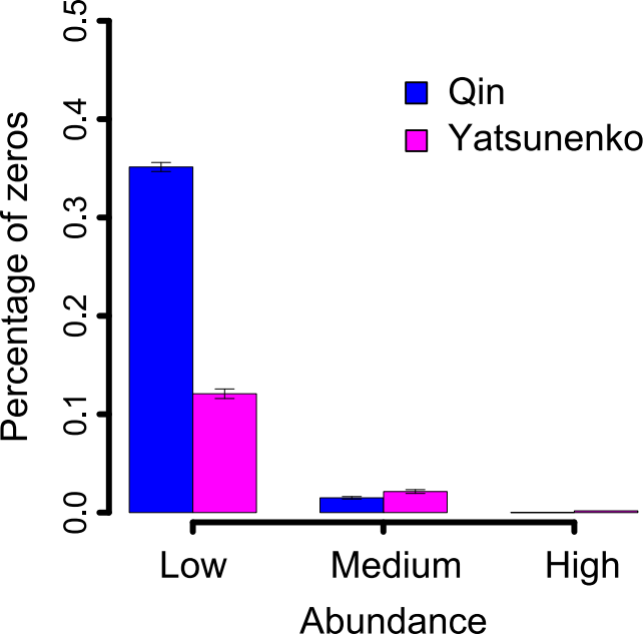

Supplement: Additional file 14: Figure S9. — The abundance of zeroes averaged over resampled data sets. There is a large difference in the number of zeros for the low abundant genes but small differences for the more highly abundant genes. The cutoffs in average number of DNA fragments were the same as used in previous results, i) ≤500, ii) 500–5000 and iii) >5000 for the Qin dataset and e i) ≤10, ii) 10–50 and iii) >50 for the Yatsunenko dataset. The group size was fixed to 6 + 6. Error bars indicate the 95 % confidence interval for the mean between resampled data sets. (PDF 19 kb) [file 12864_2016_2386_MOESM14_ESM.pdf]
