# Supplementary material for: Statistical evaluation of methods for identification of differentially abundant genes in comparative metagenomics
Source: BMC Genomics. 2016 Jan 25;17:78. doi: 10.1186/s12864-016-2386-y (PMC4727335; doi:10.1186/s12864-016-2386-y)
Supplement: Additional file 5: Table S2. — The gene ranking performance at different effect sizes for all 14 methods. Each listed value is the normalized area under curve up until a false positive rate of 0.05. Higher values represent higher gene ranking performance. The results are calculated based on 100 resampled metagenomes. The Wilcoxon-Mann–Whitney test was not evaluated at the smallest sample size (3 + 3) due to lack of samples. The full area under curve (AUC) is available in Table S3. (DOCX 13 kb) [file 12864_2016_2386_MOESM5_ESM.docx]

**Table S2. The gene ranking performance at different effect sizes for all 14 methods.**

| **AUC_0.05_** | **Data set 1: (Qin 2010)** | | | **Data set 2: (Yatsunenko 2012)** | | |
| --- | --- | --- | --- | --- | --- | --- |
| **Effect size** | **3** | **5** | **7** | **3** | **5** | **7** |
| **edgeR** | 0.68 | 0.77 | 0.81 | 0.63 | 0.80 | 0.87 |
| **DESeq2** | 0.71 | 0.80 | 0.84 | 0.63 | 0.78 | 0.84 |
| **OGLM** | 0.74 | 0.83 | 0.86 | 0.59 | 0.73 | 0.79 |
| **MetagenomeSeq** | 0.54 | 0.68 | 0.74 | 0.55 | 0.70 | 0.78 |
| **Metastats** | 0.66 | 0.74 | 0.77 | 0.52 | 0.66 | 0.74 |
| **Voom** | 0.67 | 0.74 | 0.77 | 0.56 | 0.70 | 0.76 |
| **Sqrt t-test** | 0.70 | 0.78 | 0.81 | 0.57 | 0.70 | 0.77 |
| **Log t-test** | 0.69 | 0.76 | 0.79 | 0.56 | 0.69 | 0.75 |
| **t-test** | 0.69 | 0.77 | 0.79 | 0.55 | 0.67 | 0.72 |
| **Welch t-test** | 0.66 | 0.74 | 0.76 | 0.52 | 0.62 | 0.67 |
| **WMW** | 0.67 | 0.75 | 0.78 | 0.53 | 0.66 | 0.72 |
| **Binomial** | 0.37 | 0.46 | 0.48 | 0.38 | 0.51 | 0.58 |
| **GLM** | 0.31 | 0.35 | 0.34 | 0.39 | 0.52 | 0.58 |
| **Fisher’s exact test** | 0.30 | 0.35 | 0.34 | 0.38 | 0.51 | 0.58 |

Each listed value is the normalized area under curve up until a false positive rate of 0.05. Higher values represent higher gene ranking performance. The results are calculated based on 100 resampled metagenomes. The Wilcoxon-Mann-Whitney test was not evaluated at the smallest sample size (3+3) due to lack of samples. The full area under curve (AUC) is available in S4 Table.
