# Supplementary material for: Statistical evaluation of methods for identification of differentially abundant genes in comparative metagenomics
Source: BMC Genomics. 2016 Jan 25;17:78. doi: 10.1186/s12864-016-2386-y (PMC4727335; doi:10.1186/s12864-016-2386-y)
Supplement: Additional file 13: Table S5. — Summary of results from the FDR analysis. The median value for the number of true positives detected, the number of false positives detected and achieved true FDR at a cutoff of 0.05 estimated FDR for both data sets. The group sizes were set to 6 + 6 and the effect size to 5. The results were based on 100 resampled metagenomes. (DOCX 15 kb) [file 12864_2016_2386_MOESM13_ESM.docx]

**Table S5. Summary of results from the FDR analysis.**

| **FDR Results** | **Data set 1: (Qin 2010)** | | | **Data set 2: (Yatsunenko 2012)** | | |
| --- | --- | --- | --- | --- | --- | --- |
|  | **#TRUE** | **#FALSE** | **FDR** | **#TRUE** | **#FALSE** | **FDR** |
| **edgeR** | 106 | 5 | 0.044 | 100 | 4 | 0.043 |
| **DESeq2** | 114 | 5 | 0.046 | 96 | 5 | 0.049 |
| **OGLM** | 113 | 4 | 0.034 | 80 | 3 | 0.031 |
| **mSeq** | 124 | 36 | 0.234 | 86 | 5 | 0.060 |
| **Metastats** | 103 | 4 | 0.034 | 54 | 1 | 0.021 |
| **voom** | 96 | 2 | 0.019 | 73 | 1 | 0.014 |
| **sqrtT** | 106 | 3 | 0.027 | 69 | 1 | 0.016 |
| **logT** | 104 | 3 | 0.026 | 70 | 2 | 0.023 |
| **tTest** | 96 | 2 | 0.020 | 58 | 1 | 0.020 |
| **welch** | 78 | 1 | 0.012 | 35 | 0 | 0.000 |
| **WMW** | 101 | 1 | 0.014 | 0 | 0 | 0.000 |
| **binomial** | 149 | 1229 | 0.892 | 141 | 436 | 0.755 |
| **GLM** | 149 | 1229 | 0.892 | 143 | 448 | 0.760 |
| **Fisher** | 149 | 1229 | 0.892 | 141 | 436 | 0.756 |

The median value for the number of true positives detected, the number of false positives detected and achieved true FDR at a cutoff of 0.05 estimated FDR for both data sets. The group sizes were set to 6+6 and the effect size to 5. The results were based on 100 resampled metagenomes.
