# Supplementary material for: Statistical evaluation of methods for identification of differentially abundant genes in comparative metagenomics
Source: BMC Genomics. 2016 Jan 25;17:78. doi: 10.1186/s12864-016-2386-y (PMC4727335; doi:10.1186/s12864-016-2386-y)
Supplement: Additional file 15: Table S6. — Area under curve estimates on unfiltered data for all 14 methods. Higher values represent higher gene ranking performance. The results are calculated based on 100 resampled metagenomes. The Group size was 6 + 6 and the effect fixed to 5. (DOCX 12 kb) [file 12864_2016_2386_MOESM15_ESM.docx]

**Table S6. Area under curve estimates on unfiltered data for all 14 methods.**

|  | **AUC_0.05_** | | **AUC** | |
| --- | --- | --- | --- | --- |
|  | **Data set 1:  (Qin 2010)** | **Data set 2: (Yatsunenko 2012)** | **Data set 1:  (Qin 2010)** | **Data set 2: (Yatsunenko 2012)** |
| **edgeR** | 0.68 | 0.49 | 0.91 | 0.81 |
| **DESeq2** | 0.71 | 0.46 | 0.88 | 0.78 |
| **OGLM** | 0.73 | 0.43 | 0.93 | 0.79 |
| **MetagenomeSeq** | 0.05 | 0.12 | 0.89 | 0.79 |
| **Metastats** | 0.65 | 0.39 | 0.84 | 0.79 |
| **voom** | 0.64 | 0.43 | 0.85 | 0.78 |
| **Sqrt t-test** | 0.66 | 0.41 | 0.88 | 0.78 |
| **Log t-test** | 0.66 | 0.40 | 0.84 | 0.77 |
| **t-test** | 0.67 | 0.40 | 0.90 | 0.79 |
| **Welch t-test** | 0.64 | 0.37 | 0.90 | 0.78 |
| **WMW** | 0.63 | 0.40 | 0.84 | 0.77 |
| **binomial** | 0.43 | 0.31 | 0.85 | 0.72 |
| **GLM** | 0.35 | 0.32 | 0.85 | 0.74 |
| **Fisher’s exact test** | 0.35 | 0.31 | 0.85 | 0.72 |

Higher values represent higher gene ranking performance. The results are calculated based on 100 resampled metagenomes. The Group size was 6+6 and the effect fixed to 5.
